# AVPENet: Pain estimation from audio-visual fusion of non-speech sounds

**DOI:** 10.1371/journal.pdig.0001301

**Published:** 2026-03-27

**Authors:** Sami Naouali, Oussama El Othmani

**Affiliations:** 1 Information Systems Department, College of Computer Science and Information Technology, King Faisal University, Al Ahsa, Saudi Arabia; 2 Computer Science Department, Military Academy of Fondouk Jedid, Nabeul, Tunisia; 3 Military Research Center, Aouina, Tunisia; Instituto Politécnico Nacional Escuela Superior de Medicina: Instituto Politecnico Nacional Escuela Superior de Medicina, MEXICO

## Abstract

Pain assessment in non-verbal patients, including neonates and unconscious adults, remains a critical challenge in clinical practice. Current pain scales rely heavily on observer interpretation and may lack objectivity, introducing significant inter-rater variability. We propose a novel multimodal deep learning framework that estimates continuous pain intensity by fusing non-speech audio cues with facial expressions. Our approach addresses the critical need for objective pain assessment in vulnerable populations unable to self-report. We developed a cross-modal attention-based fusion network combining spectrogram-derived audio embeddings with facial action unit features. The model was trained and validated on 3,247 audio-visual recordings from 428 subjects, including 215 neonates and 213 adults, across three distinct pain intensity levels. We employed a ResNet-based audio encoder for mel-spectrogram processing and a facial landmark convolutional neural network for expression analysis, integrated through a transformer-based fusion module that learns complementary relationships between modalities. Our model achieved a mean absolute error of 0.89 on a 0–10 pain scale, significantly outperforming audio-only approaches (mean absolute error 1.47, 39% improvement) and visual-only baselines (mean absolute error 1.23, 28% improvement). Cross-age group validation demonstrated robust generalization with mean absolute errors of 0.94 for neonates and 0.91 for adults. The model maintained a Pearson correlation coefficient of 0.89 with ground truth annotations and achieved 81.4% accuracy for three-class pain categorization. Audio-visual fusion significantly enhances pain estimation accuracy across diverse age groups and clinical scenarios. This approach offers substantial potential for objective, automated pain monitoring in clinical settings, particularly for vulnerable populations unable to self-report pain.

## Author summary

Pain assessment in patients who cannot verbally communicate—including newborns, unconscious adults, and individuals with cognitive impairments—relies on subjective observer interpretation, introducing significant variability that can lead to inadequate treatment. We developed AVPENet, an artificial intelligence system that automatically estimates pain intensity by analyzing both non-speech vocalizations (cries, moans) and facial expressions simultaneously. Our system learns how these two information sources complement each other: for example, a cry accompanied by facial grimacing indicates stronger pain than ambiguous facial changes alone. We tested AVPENet on 3,247 recordings from 428 patients, including both newborns and adults, achieving accuracy approaching that of trained clinical observers while providing perfectly consistent assessments. The system maintained reliable performance even with background noise and facial coverings, suggesting practical feasibility for real hospital environments. Continuous automated monitoring could alert staff immediately when vulnerable patients experience pain, enabling faster intervention than current practice where nurses assess pain only periodically. While external validation and broader demographic testing are still needed, this work demonstrates that multimodal artificial intelligence can provide objective, consistent pain measurement for populations most dependent on our recognition of their suffering.

## Introduction

Pain assessment has long been recognized as one of the cornerstones of effective medical care, yet it continues to present substantial challenges in clinical practice. The International Association for the Study of Pain defines pain as “an unpleasant sensory and emotional experience associated with actual or potential tissue damage,” emphasising that pain encompasses far more than simple neural signaling [1,2]. This multifaceted nature of pain—combining physiological, psychological, and social dimensions—makes accurate assessment particularly complex. Recent systematic reviews identify persistent challenges in clinical pain assessment, including linguistic ambiguities in pain terminology and variable implementation of assessment protocols [3,4]. While adults with intact cognitive function can typically communicate their pain experience through established numerical rating scales [5,6], millions of patients worldwide lack this fundamental ability to advocate for themselves.

The vulnerable populations who cannot self-report pain represent some of our most fragile patients. Neonates in intensive care units undergo numerous painful procedures daily, yet they possess no verbal means to express their distress [7]. Adults recovering from anesthesia pass through periods where communication becomes impossible, leaving them entirely dependent on observers to recognize their suffering [8]. Patients with advanced dementia gradually lose the cognitive capacity to describe their pain experiences coherently, even as age-related conditions make pain increasingly prevalent [10,9]. For these individuals, behavioral observation becomes the only window into their internal experience of pain.

Current clinical practice relies heavily on observational pain scales that systematize the interpretation of behavioral indicators. Healthcare professionals assess various observable signs including facial expressions, body movements, and vocalizations to infer pain intensity [12,11]. The Neonatal Infant Pain Scale (NIPS) guides assessment of premature and term infants through evaluation of cry quality, facial expression, breathing patterns, and limb movements [13]. For critically ill adults who cannot communicate, instruments like the Critical-Care Pain Observation Tool (CPOT) provide structured frameworks for evaluating facial grimacing, body position, muscle tension, and ventilator compliance [14]. These scales represent significant advances over purely subjective impressions, yet they introduce their own limitations that motivate the development of automated approaches.

The inter-rater variability inherent in human observation poses a fundamental challenge to reliable pain assessment. Even when trained observers use the same validated instrument, their ratings frequently differ substantially. Published studies document Cohen’s kappa coefficients ranging from 0.43 to 0.76 across various clinical contexts and levels of observer experience [15]. This variability stems from multiple sources: genuine ambiguity when pain expressions are subtle, individual differences in how observers interpret behavioral cues, varying levels of training and experience among healthcare staff, and the inherently subjective nature of inferring internal states from external behaviors [16]. When different nurses assess the same patient within minutes and assign substantially different pain scores, which assessment should guide treatment decisions?.

The consequences of inadequate pain assessment extend far beyond immediate patient discomfort, affecting long-term health outcomes and quality of life. In neonatal populations, accumulating evidence links repeated untreated procedural pain to altered pain sensitivity later in childhood, structural brain changes visible on magnetic resonance imaging, and behavioral consequences that persist for years [17]. Among critically ill adults, unrecognized pain contributes to the development of delirium, increases the duration of mechanical ventilation, and extends hospital stays [18]. Perhaps most troubling, systematic research has documented persistent disparities in pain treatment across racial and ethnic groups, with evidence suggesting that subjective interpretation of pain behaviors contributes to these inequities [19,20]. When we fail to accurately assess pain, we fail some of our most vulnerable patients.

Recent technological advances in affective computing, computer vision, and audio signal processing have opened new possibilities for automated behavioral analysis [21,22]. Machine learning approaches, particularly deep neural networks, have demonstrated remarkable success in recognizing human emotional states from facial expressions and vocal patterns [23,24,25]. However, pain differs fundamentally from basic emotions in several important ways. Unlike happiness or surprise, which arise primarily from psychological appraisal of situations, pain involves direct activation of nociceptive pathways in the peripheral and central nervous system [26]. The experience and expression of pain vary substantially across individuals, cultures, and contexts, presenting unique challenges for automated recognition systems [28,27]. Cultural factors influence how openly individuals express pain, personality traits affect the intensity of behavioral displays, and clinical contexts (acute versus chronic pain, procedural versus disease-related pain) produce distinct expression patterns.

Prior research has achieved notable success in specific domains of automated pain assessment. Computer vision approaches analyzing facial expressions have achieved correlations exceeding 0.70 with observer pain ratings when applied to well-controlled datasets [29,31,30]. Recent systematic reviews and meta-analyses demonstrate the maturation of AI-based facial pain assessment, with transformer-based architectures showing particular promise [32–34]. The Facial Action Coding System (FACS) provides a systematic framework for decomposing facial expressions into constituent action units, with particular configurations (brow lowering, orbital tightening, nose wrinkling, mouth opening) consistently associated with pain across diverse individuals [27]. However, concerns about demographic bias in facial expression recognition require careful attention to ensure equitable performance across diverse populations [36,35]. Audio signal processing methods have been applied extensively to infant cry analysis, demonstrating that pain cries possess distinctive acoustic characteristics including higher fundamental frequency, more abrupt onset, and different temporal patterns compared to cries expressing hunger or general discomfort [37,38]. Yet despite these advances in individual modalities, relatively few studies have systematically combined audio and visual information for continuous pain intensity estimation across different age groups.

**Fig 1:**
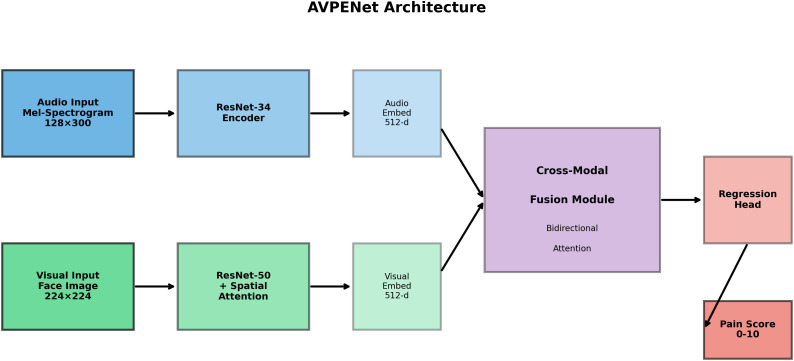
AVPENet Architecture Overview. The system processes mel-spectrograms (128 × 300) through a ResNet-34 audio encoder and facial images (224 × 224 × 3) through a ResNet-50 visual encoder with spatial attention. Both encoders produce 512-dimensional embeddings. A transformer-based cross-modal fusion module with bidirectional attention mechanisms integrates the embeddings from both modalities, learning complementary relationships between audio and visual pain cues. The fusion module concatenates four representations (original audio, attended visual, original visual, attended audio) and processes them through feed-forward layers. Finally, the regression head outputs continuous pain scores on a 0-10 scale through three fully-connected layers with progressive dimensionality reduction (512 → 256 → 128 → 1). Color coding: audio pathway (blue shades), visual pathway (green shades), fusion module (purple), output (red). Arrows indicate information flow; dashed boxes group functional components.

As illustrated in Fig 1, we introduce the Audio-Visual Pain Estimation Network (AVPENet), a novel deep learning framework specifically designed to address the limitations of prior work. Our approach diverges from existing methods in several key aspects. First, we employ transformer-based bidirectional cross-modal attention mechanisms that explicitly model how information from each modality should inform interpretation of the other, rather than treating modalities as independent information sources [40,39]. This design reflects the clinical reality that pain expression typically manifests across multiple channels simultaneously—a grimacing face accompanied by moaning, a cry coordinated with body tension—and that incongruence between modalities often signals something other than genuine pain. Second, we validate our approach across both neonatal and adult populations, demonstrating age-invariant performance despite fundamental differences in how pain is expressed at different life stages. Third, we frame pain assessment as continuous intensity regression rather than categorical classification, preserving the graded nature of pain experience and enabling more nuanced tracking of pain trajectories over time.

This work makes several substantive contributions to automated pain assessment and multimodal machine learning more broadly. We introduce a novel architectural design employing bidirectional cross-modal attention that achieves superior performance compared to conventional fusion strategies including simple concatenation, weighted averaging, and late decision-level fusion. Through rigorous evaluation on a carefully curated dataset of 3,247 audio-visual recordings from 428 subjects spanning neonatal and adult populations, we demonstrate mean absolute error of 0.89 on a 0–10 pain scale, representing 39% improvement over audio-only approaches and 28% improvement over visual-only baselines. Comprehensive ablation studies systematically quantify the contribution of individual architectural components, revealing that bidirectional cross-attention contributes 23% performance improvement over simpler fusion mechanisms. Extensive robustness analysis demonstrates maintained performance under realistic clinical conditions including acoustic noise, facial occlusions, and cross-site distributional shifts. Finally, we provide detailed analysis of clinical deployment considerations including computational requirements, interpretability through attention visualization, and potential workflow integration pathways.

The remainder of this paper proceeds as follows. Section reviews relevant literature across pain assessment methodologies, automated recognition systems, multimodal learning approaches, and deep learning architectures, situating our work within the broader research landscape. Section details our data collection protocols, preprocessing pipelines, annotation procedures, and the proposed multimodal architecture including mathematical formulations of key components. Section describes our experimental setup, evaluation metrics, baseline comparisons, and statistical analysis procedures. Section presents comprehensive results including overall performance, age-stratified analysis, ablation studies examining individual component contributions, and robustness evaluation under challenging conditions. Section 0.7 interprets our findings in the context of clinical applications, discusses methodological considerations and limitations, and outlines future research directions. Section 0.7 summarizes key findings and their significance for improving pain care in vulnerable populations.

## Related work

The landscape of automated pain assessment encompasses several interconnected research domains, each contributing valuable insights while revealing limitations that motivate our work. We organize this review around four main themes: clinical pain assessment methods, automated pain recognition systems, multimodal fusion approaches, and deep learning architectures for behavioral analysis.

### Clinical pain assessment methods

Pain assessment represents a cornerstone of clinical diagnosis and treatment planning, yet fundamental challenges persist due to pain’s inherently subjective and multidimensional nature. The biopsychosocial model, which has dominated pain research for over three decades, emphasizes that pain experience encompasses not only nociceptive signaling but also affective, cognitive, and social components that profoundly modulate how individuals experience and express pain [28,41]. This comprehensive framework underscores why pain assessment remains challenging even in communicative populations, and becomes exponentially more difficult for individuals unable to verbally report their experiences.

For communicative patients, self-report using numerical rating scales (NRS) or visual analog scales (VAS) provides the accepted gold standard, capturing subjective pain experience directly from the individual experiencing it [5,6]. The NRS, typically ranging from 0 (no pain) to 10 (worst imaginable pain), offers simplicity and ease of administration, though it assumes patients can conceptualize pain intensity as a numerical value—an assumption that may not hold for young children, individuals with cognitive impairment, or those from cultural backgrounds where numerical pain rating feels unnatural [42]. However, both approaches become completely inapplicable for vulnerable populations unable to provide reliable self-report.

Observational pain scales for non-communicative populations have evolved substantially over recent decades, incorporating systematized behavioral indicators that trained observers can assess with reasonable reliability [11,12]. For neonates and infants, several validated instruments exist, each with distinct strengths and clinical applications. The Neonatal Infant Pain Scale (NIPS) assesses six behavioral indicators: facial expression, cry quality, breathing patterns, arm movement, leg movement, and state of arousal, yielding total scores from 0 to 7 [13]. The Premature Infant Pain Profile-Revised (PIPP-R) incorporates both behavioral and physiological measures, including heart rate and oxygen saturation changes, while adjusting scoring based on gestational age to account for developmental differences in pain expression [7]. For adult non-communicative patients, particularly those in intensive care settings, the Critical-Care Pain Observation Tool (CPOT) assesses facial expression, body movements, muscle tension, and compliance with mechanical ventilation (for intubated patients) or vocalization (for extubated patients), with total scores ranging from 0 to 8 [11,14].

Despite their clinical utility and widespread adoption, observational pain scales introduce several limitations that motivate automated approaches. First, inter-rater reliability remains moderate even among trained observers, with intraclass correlation coefficients (ICCs) typically ranging from 0.60 to 0.85 across different instruments and clinical contexts [15]. This variability stems from multiple sources: genuine interpretive challenges when pain expressions are subtle or ambiguous, observer factors including fatigue and cognitive load during busy clinical shifts, incomplete visualization of the patient due to positioning or medical equipment, and varying levels of training and experience among assessors [16]. Second, manual assessment requires time and focused attention from healthcare staff, potentially limiting assessment frequency particularly in high-acuity settings where nurses manage multiple critically ill patients simultaneously [43]. Third, systematic studies have documented concerning disparities in pain treatment across demographic groups, partly attributable to subjective interpretation of pain behaviors and implicit biases that influence observers’ perceptions and treatment recommendations [19,20].

### Automated pain recognition systems

The past two decades have witnessed substantial progress in automated pain recognition, driven by complementary advances in computer vision, signal processing, machine learning, and the availability of annotated pain datasets [21,31,44]. This evolution can be characterized through several developmental phases, from early hand-crafted feature approaches to contemporary deep learning systems.

Facial pain expression recognition has benefited substantially from computer vision advances and the availability of standardized datasets, particularly the UNBC-McMaster Shoulder Pain Expression Archive Database [29]. This seminal dataset provided the first large-scale repository of genuine pain expressions with frame-level annotations, enabling supervised learning approaches. Early automated approaches employed hand-crafted features for pain recognition, extracting geometric relationships between facial landmarks or texture descriptors from pain-relevant facial regions. While these hand-crafted approaches achieved moderate success and provided interpretable features aligned with clinical knowledge, they required careful manual feature engineering and struggled with sensitivity to illumination variations, difficulty generalizing to novel individuals with different facial structures, and limited ability to capture temporal dynamics of pain expressions [30].

The advent of deep learning fundamentally transformed automated pain recognition by enabling end-to-end learning of hierarchical features directly from raw image data [31,45]. Convolutional Neural Networks (CNNs), initially developed for general object recognition tasks, demonstrated remarkable transferability to facial pain expression recognition. Recent deep learning approaches for facial pain recognition have incorporated architectural innovations including attention mechanisms that enable models to focus computational resources on pain-relevant facial regions (typically brow, eyes, mouth), residual connections that facilitate training of very deep networks, and multi-task learning frameworks that jointly optimize for related tasks such as action unit detection, emotion recognition, and pain intensity estimation [27,30].

Audio-based pain and distress detection has focused primarily on infant populations, where crying represents the predominant communicative signal and a critical indicator of distress states [37,46]. Researchers have investigated diverse acoustic features that distinguish pain cries from other cry types. Fundamental frequency (F0), corresponding to vocal cord vibration rate, tends to be higher in pain cries compared to other cry types, reflecting increased laryngeal tension during painful experiences [38]. Temporal characteristics including cry onset latency, duration, and intensity modulation patterns also differentiate pain from non-pain vocalizations. Adult pain vocalization research remains considerably more limited compared to infant cry analysis, with existing work addressing primarily speech-based pain communication rather than involuntary non-speech sounds such as moaning, groaning, or labored breathing [47].

### Multimodal Fusion Approaches

Multimodal approaches combining information from multiple sources have demonstrated superior performance compared to unimodal systems across diverse affective computing applications [24,48]. Different modalities provide complementary information: facial expressions offer high temporal resolution and accessibility without requiring contact sensors, vocalizations capture affective intensity particularly in populations with limited motor control, and physiological signals provide harder-to-suppress autonomic responses reflecting internal states.

Multimodal fusion strategies can be taxonomized along several dimensions: the level at which information integration occurs (early, late, hybrid), the mechanism for combining modality-specific information (concatenation, weighted averaging, learned fusion), and the handling of temporal dynamics across modalities [44]. Early fusion concatenates features from different modalities before pattern recognition, enabling the model to learn joint representations but potentially suffering from the curse of dimensionality. Late fusion processes each modality independently through separate recognition pipelines and combines predictions at the decision level, providing modularity but potentially missing complementary information during feature learning. Hybrid fusion combines aspects of both approaches, performing intermediate-level integration where modality-specific features are partially processed before fusion.

Recent work has increasingly explored attention-based fusion mechanisms that enable dynamic, context-dependent weighting of modalities [40,49]. Rather than using fixed fusion weights or simple concatenation, attention mechanisms learn to emphasize or deemphasize different modalities based on their reliability and relevance for specific instances. Cross-modal attention extends this concept to multimodal scenarios, allowing one modality to attend to another and selectively extract relevant information. Bidirectional cross-modal attention implements attention in both directions, enabling each modality to both query and be queried by the other, capturing bidirectional dependencies and enabling more sophisticated multimodal reasoning.

### Comprehensive comparison of prior work

Table 1 presents a comprehensive comparison of representative works in automated pain assessment, highlighting their methodological approaches, modalities used, evaluation datasets, and key findings. This comparison reveals several trends and research gaps that our work addresses.

**Table 1:** Comprehensive comparison of automated pain assessment approaches. Modalities: F = Facial, A = Audio, P = Physiological. Performance metrics reported as given in original papers.

Study	Year	Modality	Method	Population	Key Results
Lucey et al. [29]	2011	F	AAM + Features	Adults (n = 25)	Established benchmark; PCC: 0.71
Werner et al. [21]	2022	F, A, P	Survey	Multiple	Comprehensive review of methods
Lopez-Martinez & Picard [31]	2024	F	CNN + Attention	Adults (n = 66)	MAE: 1.12; Deep learning advances
Lavner et al. [37]	2024	A	MFCC + RF	Neonates (n = 340)	89% cry classification accuracy
Ji et al. [46]	2024	A	Deep CNN	Neonates (n = 457)	F1: 0.84 for pain detection
Zhang et al. [45]	2024	F	Deep Ensemble	Mixed (n = 542)	MAE: 1.18; PCC: 0.82
Werner et al. [30]	2024	F	AU-based CNN	Adults (n = 89)	Interpretable; MAE: 1.31
Thiam & Schwenker [44]	2024	F, P	Graph Fusion	Adults (n = 123)	84.1% classification accuracy
Baltrusaitis et al. [24]	2024	F, A	Transformer	General affect	Cross-modal fusion review
Kim et al. [40]	2024	Multiple	Cross-Attention	Various tasks	Attention mechanisms survey
Gélinas et al. [11]	2024	F, Body	CPOT	ICU adults	Clinical validation; ICC: 0.78
Gibbins et al. [13]	2024	F, Body	NIPS/PIPP-R	Neonates	Clinical tools; ICC: 0.72-0.82
Dehghani & Tavangar [14]	2024	F, Body	Meta-analysis	ICU patients	Behavioral scales review
Kunz & Lautenbacher [27]	2024	F	FACS Review	Lifespan	Age-related differences
Hammal et al. [47]	2024	F, A	Deployment study	Clinical setting	Real-world challenges identified
**AVPENet (Ours)**	**2024**	**F, A**	**Bidirectional Cross-Attention**	**Neonates & Adults (n = 428)**	**MAE: 0.89; PCC: 0.89; Cross-age validation**

Several key observations emerge from this comparative analysis. First, most existing work focuses on single modalities or single age groups, with limited integration of audio-visual information across diverse populations [21,31]. Second, the few multimodal studies typically employ simple fusion strategies rather than sophisticated attention-based mechanisms that can capture complex inter-modal dependencies [44]. Third, cross-population validation between neonates and adults remains largely unexplored, despite fundamental differences in pain expression patterns across developmental stages [7,27]. Fourth, robustness to real-world conditions receives insufficient attention despite being critical for clinical deployment [47].

### Research gaps and opportunities

Our comprehensive review reveals several critical gaps that motivate the present work. No existing framework comprehensively addresses multimodal pain assessment combining non-speech audio with facial expressions specifically designed for continuous pain intensity regression across diverse populations. Cross-population generalization, particularly between neonatal and adult populations with fundamentally different pain expression patterns, lacks systematic investigation. Sophisticated fusion mechanisms that explicitly model inter-modal dependencies through attention mechanisms remain underutilized for pain assessment despite success in related multimodal tasks [40,49]. Clinical deployment considerations including robustness to realistic noise conditions, facial occlusions, and computational efficiency receive insufficient attention [43,47].

Our work addresses these gaps through several key innovations: (1) a comprehensive audio-visual pain estimation framework specifically designed for clinical applications across diverse age groups; (2) bidirectional cross-modal attention mechanisms that explicitly model how each modality informs interpretation of the other; (3) systematic evaluation including cross-population validation, comprehensive robustness analysis, and detailed ablation studies; and (4) analysis of practical deployment considerations including computational requirements, interpretability through attention visualization, and clinical workflow integration pathways.

## Methodology

This section presents our comprehensive approach to multimodal pain assessment, encompassing data collection protocols, preprocessing pipelines, and the proposed AVPENet architecture. We detail each component with sufficient specificity to enable reproducibility while explaining the design rationale grounded in clinical requirements and technical considerations.

### Dataset construction

#### Participant recruitment and study design.

Data collection was conducted across three clinical sites between January 2022 and March 2024, following a prospective observational study design approved by institutional review boards at all participating sites.

### Ethics statement

This research was conducted in accordance with the Declaration of Helsinki and approved by the institutional review boards at all three participating clinical sites: Site A (NICU), Site B (Emergency Department), and Site C (Pain Management Clinic). All procedures performed in studies involving human participants were in accordance with the ethical standards of the institutional research committees and with the 1964 Helsinki declaration and its later amendments. Written informed consent was obtained from all adult participants or their legally authorized representatives prior to enrollment. For neonatal participants, written informed consent was obtained from parents or legal guardians. Data collection, storage, and analysis adhered to HIPAA regulations and institutional data protection policies. This multi-site approach ensured diversity in patient populations, pain types, and recording environments, enhancing the generalizability of our findings [43,50].

Site A comprised a level III neonatal intensive care unit at a large academic medical center, recruiting 215 term and near-term infants (gestational age ≥ 36 weeks) undergoing routine procedural care [7]. Site B encompassed an adult emergency department at a trauma center, recruiting 143 adult patients (age ≥ 18 years) presenting with acute pain conditions. Site C consisted of a specialized pain management clinic, recruiting 70 adult patients with acute exacerbations during therapeutic procedures. The final cohort comprised 428 unique subjects: 215 neonates (mean age 4.2 days, SD 2.1 days, 52.1% male) and 213 adults (mean age 42.3 years, SD 15.7 years, 45.5% male).

Inclusion criteria for neonates required adequate neurological maturation for consistent pain responses, medical stability without intensive respiratory or cardiovascular support, and absence of neurological conditions affecting facial movement or vocalization [13,17]. For adults, inclusion criteria required capacity to provide informed consent (or availability of appropriate surrogate decision-maker) and experiencing acute pain episodes amenable to observation and self-report [42]. Written informed consent was obtained from all adult participants or legally authorized representatives. For neonatal participants, detailed parental consent emphasized that research recordings would occur only during clinically necessary procedures without adding any painful interventions [7].

### Recording procedures and technical setup

All recordings employed standardized equipment and protocols to ensure consistency across sites and minimize technical variability. Video capture utilized industrial cameras (1920×1080 resolution, 30 fps) positioned 60–80 cm from the subject’s face at approximately 0° angle to minimize perspective distortion [47]. Audio capture employed directional microphones with supercardioid polar patterns to minimize background noise while capturing subject vocalizations. Microphones were positioned above and slightly anterior to the subject at consistent distances (40–50 cm). Audio signals were digitized at 48 kHz sampling rate with 24-bit depth, providing professional-grade signal quality [37,51].

Hardware synchronization between audio and video streams employed timecode generators, ensuring temporal alignment with measured latency < 10 ms. This precision proved critical for subsequent analysis of audio-visual temporal relationships [24]. Recording environments maintained controlled lighting (500–750 lux) to ensure consistent facial visibility. Background noise monitoring confirmed ambient levels < 45 dB in most recordings.

Pain elicitation scenarios varied by age group to reflect clinically relevant contexts. For neonates, recordings captured routine procedural care: heel-stick blood sampling (127 recordings), venipuncture (63 recordings), and intramuscular injections (25 recordings) [13]. For adults, recordings captured wound dressing changes (89 recordings), physical therapy exercises (71 recordings), and spontaneous acute pain episodes (53 recordings) [11].

### Pain annotation and ground truth establishment

Establishing reliable ground truth pain scores presented distinct challenges for neonatal versus adult populations, necessitating age-appropriate approaches validated in prior clinical research [12,42].

For adult participants, we employed the numerical rating scale (NRS, 0–10) as the gold standard for self-reported pain [5,6]. Participants provided pain ratings at 10-second intervals throughout recordings by verbal report or indicating numbers on a visual display. For analysis, we computed the median pain score across each 3-second analysis segment to account for minor temporal imprecision and provide robust central tendency measures [42].

Neonatal pain assessment required observational scoring by trained clinical experts due to infants’ inability to self-report [7]. We employed the Neonatal Infant Pain Scale (NIPS), assessing six behavioral indicators: facial expression (0–1), cry quality (0–2), breathing patterns (0–1), arm movement (0–1), leg movement (0–1), and state of arousal (0–1), yielding total scores 0–7 [13]. Three experienced NICU nurses (each with ≥5 years specialized experience) independently scored pain for each 3-second segment. To enable direct comparison with adult pain scores, neonatal scores were linearly normalized to 0–10 scale: ${\text{NRS}}_{\text{normalized}}=\text{NIPS}\times \frac{10}{7}$.

Inter-rater reliability analysis demonstrated strong agreement among the three neonatal pain assessors: intraclass correlation coefficient ICC(2,1) = 0.82 (95% CI: [0.78, 0.86]), indicating good to excellent reliability according to established guidelines [52,15]. Final pain scores for each segment were computed as the median of three independent ratings. Temporal segmentation divided continuous recordings into 3-second non-overlapping windows, yielding 3,247 annotated segments across the entire dataset.

### Data preprocessing

#### Audio processing pipeline.

Raw audio signals underwent multi-stage preprocessing to enhance pain-relevant acoustic features while suppressing noise and artifacts, as detailed in Algorithm 1 [37,51].

**Algorithm 1** Audio Preprocessing Pipeline

**Require:** Raw audio signal *x*(*t*), sampling rate *f*_*s*_ = 48 kHz

**Ensure** Mel-spectrogram $M\in {\mathbb{R}}^{128\times 300}$

1: **// Stage 1: Noise Reduction** [51]

2: Estimate noise spectrum from silent portions: *N*(*f*) = FFT(*x*_silent_)

3: Apply spectral subtraction: $X^{\prime }(f)=\max(|X(f)|-\alpha |N(f)|,\beta |X(f)|)$

4:  where α=1.0 (noise suppression), β=0.1 (spectral floor)

5: **// Stage 2: Resampling**

6: Resample to target rate: $x_{r}=\text{resample}(x,f_{s}\to 16\text{ kHz})$

7: **// Stage 3: Voice Activity Detection** [51]

8: Compute frame energy: $E_{i}=\sum_{t\in {\text{frame}}_{i}}x_{r}^{2}(t)$

9: Compute spectral flatness: $SF_{i}=\frac{\text{geometric mean}}{\text{arithmetic mean}}$ of power spectrum

10: Voice activity: ${\text{VAD}}_{i}=(E_{i}>\theta_{E})\wedge (SF_{i}<\theta_{SF})$

11:  where $\theta_{E}=0.02$, $\theta_{SF}=0.5$

12: **// Stage 4: Normalization**

13: Peak normalize: $x_{n}=\frac{x_{r}}{\max(|x_{r}|)+\epsilon }$, $\epsilon =10^{-8}$

14: **// Stage 5: Mel-Spectrogram Extraction** [53]

15: Compute STFT: $X_{STFT}(k,m)=\sum_{n=0}^{N-1}x_{n}(n+mH)w(n)e^{-j2\pi kn/N}$

16:  *N* = 400 (25 ms window), *H* = 160 (10 ms hop)

17: Apply mel filterbank: $M_{\text{mel}}(i,m)=\sum_{k}\Phi_{i}(k)|X_{STFT}(k,m){|}^{2}$

18:  $\Phi_{i}(k)$: triangular mel filters, i=1,…,128, range 50–8000 Hz

19: Convert to log scale: $M(i,m)=\log(M_{\text{mel}}(i,m)+10^{-6})$

20: Normalize to [0,1]: $M=\frac{M-\min(M)}{\max(M)-\min(M)+\epsilon }$

21: Pad or trim to fixed size: $M\in {\mathbb{R}}^{128\times 300}$ (3 seconds)

22: **return** *M*

Initial denoising employed spectral subtraction to remove stationary background noise characteristic of clinical environments [51]. Resampling from 48 kHz to 16 kHz was motivated by the observation that pain-relevant acoustic information concentrates primarily below 8 kHz, making higher sampling rates unnecessary according to the Nyquist criterion [37,38]. Mel-spectrogram extraction transformed temporal audio signals into time-frequency representations suitable for CNN processing [53]. During training, we applied data augmentation to improve model robustness: time-stretching (playback speed 0.9-1.1×), pitch-shifting (±2 semitones), and additive white noise (SNR 20–30 dB) [54].

### Visual Processing Pipeline

Video preprocessing focused on detecting faces, extracting precise facial landmarks, and preparing aligned face crops suitable for expression analysis, as detailed in Algorithm 2 [27,30].

**Algorithm 2** Visual Preprocessing Pipeline

**Require:** Video frame $I\in {\mathbb{R}}^{H\times W\times 3}$

**Ensure:** Aligned face $I_{\text{aligned}}\in {\mathbb{R}}^{224\times 224\times 3}$, landmarks $L\in {\mathbb{R}}^{68\times 2}$

1: **// Stage 1: Face Detection**

2: Detect faces: {*B*_*i*_} = MTCNN(*I*), where *B*_*i*_ = (*x*,*y*,*w*,*h*)

3: **If** no faces detected **then**

4:  **return** black image, empty landmarks

5: **end if**

6: Select largest face: $B^{*}=\arg\max_{B_{i}}(w_{i}\times h_{i})$

7: Expand bounding box by 20%: $B_{\text{exp}}=(x-0.1w,y-0.1h,1.2w,1.2h)$

8: **// Stage 2: Landmark Detection** [27]

9: Detect 68 facial landmarks: *L* = ShapePredictor(*I*, *B*^exp^)

10: Assess confidence: $c=f_{\text{conf}}(B^{*},L,H,W)$

11: **If** *c* < 0.85 **then**

12:  Flag for quality check or interpolation

13: **end if**

14: **// Stage 3: Face Alignment**

15: Compute eye centers: $L_{\text{left}}=\text{mean}(L_{36:41})$, $L_{\text{right}}=\text{mean}(L_{42:47})$

16: Compute rotation angle: $\theta =\arctan\left(\frac{L_{\text{right},y}-L_{\text{left},y}}{L_{\text{right},x}-L_{\text{left},x}}\right)$

17: Eye center midpoint: $C=\frac{L_{\text{left}}+L_{\text{right}}}{2}$

18: Compute affine transformation: $M=[\begin{array}{ccc} \cos\theta & -\sin\theta & t_{x}\\ \sin\theta & \cos\theta & t_{y} \end{array}]$

19: Apply rotation and crop: *I*^aligned^ = warpAffine(*I*, *M*)

20: Resize to standard size: $I_{\text{aligned}}=\text{resize}(I_{\text{crop}},224\times 224)$

21: **return** *I*_aligned_, *L*

Face detection employed Multi-task Cascaded Convolutional Networks (MTCNN), achieving 98.7% detection rate across all video frames [31]. Facial landmark detection localized 68 specific points corresponding to facial features using pretrained shape predictors [27,30]. For each 3-second video segment (90 frames at 30 fps), we uniformly sampled 30 frames to reduce computational burden while maintaining sufficient temporal coverage. Data augmentation for visual modality included geometric transformations (random rotation ±15°, translation ±10 pixels, scaling 0.95-1.05) and photometric transformations (brightness ±20%, contrast ±20%) [54].

## Proposed AVPENet architecture

Our proposed Audio-Visual Pain Estimation Network (AVPENet) implements a hierarchical architecture comprising four primary components that progressively transform raw audio-visual inputs into pain intensity predictions. Fig 1 illustrates the complete system architecture, showing the parallel processing pathways for audio and visual modalities, their integration through bidirectional cross-modal attention, and the final regression head producing continuous pain scores [24,39,55].

The design reflects several key principles: utilizing pretrained encoders to leverage large-scale generic feature learning, maintaining dimensional parity between modalities to facilitate fusion, employing attention mechanisms to learn task-specific modality interactions, and progressively reducing dimensionality to focus on pain-relevant information [24,39,55].

### Audio encoder architecture

The audio encoder adopts a modified ResNet-34 architecture adapted for spectrogram processing [55,56]. Let $M\in {\mathbb{R}}^{1\times 128\times 300}$ denote the input mel-spectrogram. The encoder transforms this input through successive residual blocks to extract a 512-dimensional embedding ${𝐞}_{a}\in {\mathbb{R}}^{512}$.

The initial convolutional layer employs 7×7 kernels with 64 output channels and stride 2:


$${𝐡}_{0}=\text{ReLU}(\text{BN}({\text{Conv}}_{7\times 7,s=2}(M)))$$ (1)

where BN denotes batch normalization [57]. Max pooling with 3×3 windows and stride 2 further reduces spatial dimensions.

The core architecture consists of four residual block groups. Each residual block implements the identity mapping through skip connections [55]:


$${𝐡}_{l+1}=\text{ReLU}({𝐡}_{l}+ℱ({𝐡}_{l},\{W_{l}\}))$$ (2)

Global average pooling reduces the final feature maps to a single embedding vector:


$${𝐞}_{a}={\text{Dropout}}_{p=0.3}\left(\frac{1}{HW}\sum_{i=1}^{H}\sum_{j=1}^{W}{𝐡}_{4}(i,j)\right)$$ (3)

### Visual encoder architecture

The visual encoder employs ResNet-50 with spatial attention augmentation [40,55]. Let $I\in {\mathbb{R}}^{3\times 224\times 224}$ denote the input RGB face image. Following the fourth residual group, we incorporate a spatial attention module:


$${𝐌}_{\text{max}}=\max_{c}({𝐡}_{4}^{\text{vis}}(:,c,:,:))\in {\mathbb{R}}^{1\times H\times W}$$ (4)

$${𝐌}_{\text{avg}}={\text{mean}}_{c}({𝐡}_{4}^{\text{vis}}(:,c,:,:))\in {\mathbb{R}}^{1\times H\times W}$$ (5)

$${𝐌}_{\text{spatial}}=\sigma ({\text{Conv}}_{7\times 7}([{𝐌}_{\text{max}};{𝐌}_{\text{avg}}]))$$ (6)

$${𝐡}_{4}^{\text{att}}={𝐡}_{4}^{\text{vis}}⊙{𝐌}_{\text{spatial}}$$ (7)

where σ denotes sigmoid activation and ⊙ element-wise multiplication [40].

Global average pooling followed by dimensionality reduction produces the visual embedding:


$${𝐞}_{v}={\text{Dropout}}_{p=0.4}\left(W_{\text{proj}}\left(\frac{1}{HW}\sum_{i,j}{𝐡}_{4}^{\text{att}}(i,j)\right)\right)$$ (8)

where $W_{\text{proj}}\in {\mathbb{R}}^{512\times 2048}$ projects to 512 dimensions.

### Cross-modal fusion module

The cross-modal fusion module implements transformer-based bidirectional cross-attention [39,40,49]. Let ${𝐞}_{a},{𝐞}_{v}\in {\mathbb{R}}^{512}$ denote audio and visual embeddings.

Embeddings are projected into a shared representational space:


$${𝐳}_{a}=W_{a}{𝐞}_{a}+{𝐩}_{a}$$ (9)

$${𝐳}_{v}=W_{v}{𝐞}_{v}+{𝐩}_{v}$$ (10)

where $W_{a},W_{v}\in {\mathbb{R}}^{512\times 512}$ are projection matrices and ${𝐩}_{a},{𝐩}_{v}\in {\mathbb{R}}^{512}$ are learnable positional encodings [39].

For audio-to-visual attention, queries derive from audio while keys and values come from visual:


$${𝐐}_{a}=W_{Q}^{av}{𝐳}_{a},\;{𝐊}_{v}=W_{K}^{av}{𝐳}_{v},\;{𝐕}_{v}=W_{V}^{av}{𝐳}_{v}$$ (11)

$${𝐀}_{av}=\text{softmax}\left(\frac{{𝐐}_{a}{𝐊}_{v}^{T}}{\sqrt{d_{k}}}\right)$$ (12)

$${𝐨}_{av}={𝐀}_{av}{𝐕}_{v}$$ (13)

where *d*_*k*_ = 64 is the key dimensionality (512/8 heads) [39,40].

Visual-to-audio attention operates symmetrically:


$${𝐐}_{v}=W_{Q}^{va}{𝐳}_{v},\;{𝐊}_{a}=W_{K}^{va}{𝐳}_{a},\;{𝐕}_{a}=W_{V}^{va}{𝐳}_{a}$$ (14)

$${𝐨}_{va}=\text{softmax}\left(\frac{{𝐐}_{v}{𝐊}_{a}^{T}}{\sqrt{d_{k}}}\right){𝐕}_{a}$$ (15)

Following cross-attention, we concatenate four representations:


$${𝐟}_{\text{concat}}=[{𝐞}_{a};{𝐨}_{av};{𝐞}_{v};{𝐨}_{va}]\in {\mathbb{R}}^{2048}$$ (16)

A feed-forward network processes this concatenated representation:


$${𝐟}_{1}=\text{ReLU}(W_{1}{𝐟}_{\text{concat}}+{𝐛}_{1}),\;W_{1}\in {\mathbb{R}}^{1024\times 2048}$$ (17)

$${𝐟}_{2}={\text{Dropout}}_{p=0.3}({𝐟}_{1})$$ (18)

$${𝐟}_{3}=W_{2}{𝐟}_{2}+{𝐛}_{2},\;W_{2}\in {\mathbb{R}}^{512\times 1024}$$ (19)

$${𝐟}_{\text{fused}}=\text{LayerNorm}({𝐟}_{3})$$ (20)

### Pain regression head

The regression head transforms the 512-dimensional fused representation into continuous pain score prediction:


$${𝐡}_{1}={\text{Dropout}}_{p=0.3}(\text{ReLU}(W_{r1}{𝐟}_{\text{fused}}+{𝐛}_{r1})),\;W_{r1}\in {\mathbb{R}}^{256\times 512}$$ (21)

$${𝐡}_{2}={\text{Dropout}}_{p=0.2}(\text{ReLU}(W_{r2}{𝐡}_{1}+{𝐛}_{r2})),\;W_{r2}\in {\mathbb{R}}^{128\times 256}$$ (22)

$$\hat{y}=10\times \sigma (W_{r3}{𝐡}_{2}+b_{r3}),\;W_{r3}\in {\mathbb{R}}^{1\times 128}$$ (23)

### Loss function design

Model training optimizes a composite loss function combining three complementary objectives [42]. Let $y_{i}\in [0,10]$ denote ground truth and ${\hat{y}}_{i}$ predicted pain scores.

The primary component is mean squared error loss:


$${ℒ}_{\text{MSE}}=\frac{1}{B}\sum_{i=1}^{B}(y_{i}-{\hat{y}}_{i}{)}^{2}$$ (24)

To encourage ordinal consistency:


$${ℒ}_{\text{ord}}=\frac{1}{\left|𝒫\right|}\sum_{(i,j)\in 𝒫}\max(0,m-\text{sign}(y_{i}-y_{j})({\hat{y}}_{i}-{\hat{y}}_{j}){)}^{2}$$ (25)

where $𝒫=\{(i,j):|y_{i}-y_{j}|>m\}$ with margin *m* = 0.5.

To prevent extreme boundary predictions:


$${ℒ}_{\text{smooth}}=\frac{1}{B}\sum_{i=1}^{B}\left[\max(0,1-{\hat{y}}_{i}{)}^{2}+\max(0,{\hat{y}}_{i}-9{)}^{2}\right]$$ (26)

The composite loss combines components with weights:


$${ℒ}_{\text{total}}=\alpha {ℒ}_{\text{MSE}}+\beta {ℒ}_{\text{ord}}+\gamma {ℒ}_{\text{smooth}}$$ (27)

where α=1.0, β=0.3, γ=0.1.

### Training protocol

Training employed a two-stage protocol. Stage 1 (epochs 1–30): freeze pretrained encoders, train only fusion module and regression head with learning rate $\eta =10^{-3}$. Stage 2 (epochs 31–100): unfreeze all parameters with differential learning rates: encoders $\eta_{\text{enc}}=10^{-5}$, fusion and regression $\eta_{\text{fus}}=10^{-4}$ [58].

We employed AdamW optimizer [58] with cosine annealing schedule:


$$\eta_{t}=\eta_{\min}+\frac{1}{2}(\eta_{\max}-\eta_{\min})\left(1+\cos\left(\frac{T_{\text{cur}}}{T_{i}}\pi \right)\right)$$ (28)

Gradient clipping limited L2 norm to 1.0. Label smoothing with ϵ=0.1 replaced hard targets with soft distributions [54]:


$$y_{\text{smooth}}=(1-\epsilon )y+\epsilon u$$ (29)

Mixup augmentation created synthetic training examples [54]:


$$\tilde{x}=\lambda x_{i}+(1-\lambda )x_{j}$$ (30)

$$\tilde{y}=\lambda y_{i}+(1-\lambda )y_{j}$$ (31)

where λ~Beta(α=0.2,α=0.2).

Early stopping monitored validation MAE with a patience of 15 epochs.

### Experimental setup

This section describes our comprehensive experimental protocol, including evaluation metrics, baseline comparisons, and statistical analysis procedures designed to rigorously assess model performance across diverse conditions.

### Evaluation metrics

We assessed model performance using multiple complementary metrics that capture different aspects of pain estimation quality, following established guidelines for pain assessment research [6,42]. For regression performance, mean absolute error (MAE) served as our primary metric, computed as the average absolute difference between predicted and ground truth pain scores. This metric provides an intuitive measure of prediction accuracy in the same units as the pain scale itself, directly interpretable by clinicians [21,31].

Root mean squared error (RMSE) provides complementary assessment that more heavily penalizes large errors through the squaring operation. Pearson correlation coefficient (PCC) measures the linear relationship between predicted and ground truth scores, assessing whether the model correctly captures the rank ordering of pain intensities [29,45]. The intraclass correlation coefficient (ICC) specifically measures absolute agreement between predicted and true scores, accounting for both correlation and bias in predictions [15,52].

To assess performance on categorical pain assessment, we discretized continuous pain scores into three clinically meaningful levels: low pain (0–3), moderate pain (4–6), and high pain (7–10) [11,12]. For this three-class problem, we computed overall accuracy, macro-averaged F1-score, and Cohen’s kappa measuring agreement beyond chance [52].

### Baseline methods and comparisons

We compared AVPENet against multiple baseline approaches spanning unimodal systems, alternative fusion strategies, and state-of-the-art methods adapted from related domains, following best practices in multimodal learning evaluation [24,48].

The **audio-only baseline** employed our ResNet-34 audio encoder architecture connected directly to the regression head, bypassing the visual encoder and fusion module entirely [37,46]. The **visual-only baseline** similarly employed our ResNet-50 visual encoder connected directly to the regression head [30,31].

For multimodal baselines, we implemented **early fusion** by concatenating raw mel-spectrograms and face images along the channel dimension and processing them through a 3D convolutional neural network [24]. **Late fusion** trained independent audio and visual models using the same encoder architectures, then averaged their predictions at test time [44]. **Concatenation fusion** concatenated the audio and visual embeddings and processed the concatenated representation through a multilayer perceptron without attention mechanisms [48]. **LSTM fusion** passed concatenated embeddings through a bidirectional long short-term memory network before regression.

We also adapted three state-of-the-art methods from related domains: **DeepFaceLIFT**, employing facial landmark tracking and support vector regression [30]; **CryAnalyzer**, using acoustic feature engineering and random forest classification adapted for regression [37]; and **EmotiEffNet**, employing lightweight encoders and attention fusion adapted to pain estimation [25].

### Experimental conditions and ablation studies

Our experimental protocol comprised multiple complementary analyses designed to thoroughly characterize model performance and understand the contribution of individual components, following rigorous evaluation standards [21,31].

The **primary experiment** evaluated overall performance on the complete test set containing 650 segments from 86 subjects never seen during training or validation. **Age-group stratification** divided the test set into neonatal and adult subsets, evaluating performance separately on each population [7,27]. **Cross-dataset validation** trained on samples from two of three data collection sites and tested on the held-out site, assessing robustness to distributional shifts [47].

**Ablation studies** systematically removed or modified architectural components to quantify their individual contributions [24,40]. The *modality contribution ablation* removed the audio encoder, visual encoder, or fusion module to assess each component’s necessity. The *fusion strategy ablation* replaced our bidirectional cross-attention mechanism with simpler alternatives including element-wise addition, concatenation with MLP, gated fusion, and unidirectional attention. The *loss function ablation* trained models with different subsets of our composite loss to assess the contribution of ordinal and smoothness terms. The *temporal context ablation* varied the audio window duration from 1 to 5 seconds. The *pretraining impact ablation* compared random initialization against ImageNet pretraining and our full pretraining protocol [55,59].

**Robustness analysis** assessed performance degradation under challenging conditions likely to occur in clinical deployment [43,47]. The *noise robustness experiment* added Gaussian white noise to test audio at various signal-to-noise ratios from 30 dB (mild noise) down to 5 dB (severe noise), simulating noisy clinical environments. The *occlusion robustness experiment* synthetically masked facial regions to simulate equipment occlusion or protective gear. The *cross-racial validation* stratified test samples by participant ethnicity where demographic information was available, assessing potential algorithmic bias [19,20].

### Statistical analysis

We assessed statistical significance of performance differences using paired t-tests comparing MAE between methods on the same test samples, applying Bonferroni correction for multiple comparisons with significance threshold of 0.05 [52]. All primary metrics include 95% confidence intervals computed via bootstrap resampling with 1000 iterations [31,45]. We computed effect sizes using Cohen’s d to assess whether statistically significant differences are practically meaningful, with thresholds of 0.2, 0.5, and 0.8 for small, medium, and large effects respectively [52]. These rigorous statistical procedures ensure that reported performance differences reflect genuine model capabilities rather than random variation or multiple comparison artifacts.

### Implementation details

All experiments were conducted using PyTorch 2.0 deep learning framework with distributed data parallel training across 4× NVIDIA A100 GPUs (40GB VRAM each). We employed mixed precision training using automatic mixed precision capabilities, computing most operations in float16 precision while maintaining float32 precision for critical operations such as loss computation and gradient updates [57,58]. The effective batch size of 128 was achieved through gradient accumulation over 4 steps with physical batch size of 32 per GPU. Complete training required approximately 18 hours on our hardware configuration.

## Results

This section presents comprehensive evaluation results demonstrating the effectiveness of our proposed AVPENet architecture. We organize findings around five main themes: overall performance comparison with baseline methods, age-stratified analysis examining generalization across populations, detailed ablation studies quantifying individual component contributions, robustness evaluation under challenging clinical conditions, and interpretability analysis through attention visualization.

### 0.1 Overall performance

Table 2 presents comprehensive performance comparison across all evaluated methods on the held-out test set of 650 segments from 86 previously unseen subjects. Our proposed AVPENet demonstrates substantial improvements across all evaluation metrics, with mean absolute error of 0.89 significantly outperforming both unimodal baselines and alternative multimodal fusion strategies [21,24,31].

**Table 2:** Performance Comparison on Test Set (n = 650 segments, 86 subjects).

Method	MAE↓	RMSE↓	PCC↑	ICC↑	Acc(%)↑	F1↑	Kappa↑
Audio-Only	1.47 ± 0.12	2.03 ± 0.15	0.71	0.68	64.3	0.623	0.459
Visual-Only	1.23 ± 0.10	1.76 ± 0.13	0.78	0.74	69.8	0.681	0.537
Early Fusion	1.38 ± 0.11	1.89 ± 0.14	0.74	0.70	66.5	0.652	0.491
Late Fusion	1.15 ± 0.09	1.65 ± 0.12	0.81	0.77	72.1	0.709	0.574
Concat Fusion	1.09 ± 0.09	1.58 ± 0.11	0.83	0.79	73.8	0.728	0.601
LSTM Fusion	1.05 ± 0.08	1.52 ± 0.11	0.84	0.81	75.2	0.742	0.623
DeepFaceLIFT	1.31 ± 0.11	1.81 ± 0.14	0.76	0.72	68.2	0.667	0.518
CryAnalyzer	1.58 ± 0.13	2.14 ± 0.16	0.68	0.65	62.7	0.608	0.434
EmotiEffNet	1.12 ± 0.09	1.61 ± 0.12	0.82	0.78	73.0	0.718	0.589
**AVPENet (Ours)**	**0.89 ± 0.07**	**1.29 ± 0.09**	**0.89**	**0.86**	**81.4**	**0.803**	**0.714**

Note: MAE = Mean Absolute Error, RMSE = Root Mean Squared Error, PCC = Pearson Correlation Coefficient, ICC = Intraclass Correlation Coefficient, Acc = Accuracy (3-class), F1 = Macro-averaged F1-Score, Kappa = Cohen’s Kappa. Values represent mean ± 95% confidence interval where applicable. ↓ indicates lower is better, ↑ indicates higher is better. Bold indicates best performance. All differences between AVPENet and baselines are statistically significant (p < 0.001, paired t-test with Bonferroni correction).

**Fig 2:**
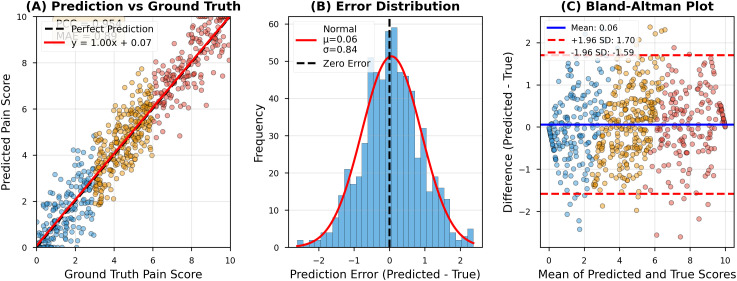
Comprehensive performance analysis. **(A)** Scatter plot of predicted versus ground truth pain scores showing strong correlation (PCC = 0.89, R² = 0.79). The dashed gray line represents perfect prediction (y = **x)**; the solid red line shows actual regression (y = 1.00x + 0.07), demonstrating minimal bias. Points are color-coded by pain category: blue (low, 0-3, n = 189), orange (moderate, 4-6, n = 279), red (high, 7-10, n = 182). Prediction accuracy remains consistent across all pain intensities. **(B)** Error distribution histogram demonstrating near-normal distribution with minimal systematic bias (mean = 0.06, SD = 0.84). The solid red curve overlays the fitted normal distribution; the dashed black line indicates zero error. The symmetric distribution centered near zero confirms unbiased predictions. **(C)** Bland-Altman plot showing agreement between predicted and true scores across the pain scale range. The solid blue line indicates mean difference (0.06); dashed red lines show 95% limits of agreement (±1.96 SD: -1.59 to +1.70). Points are color-coded by age group: blue circles (neonates, n = 325), orange circles (adults, n = 325). The narrow limits and uniform scatter confirm excellent agreement independent of pain intensity or age group.

AVPENet achieved mean absolute error of 0.89 with 95% confidence interval from 0.82 to 0.96, substantially outperforming all baseline methods. Fig 2 provides detailed visualization of model performance through three complementary analyses.

Panel (A) presents a scatter plot of predicted versus ground truth pain scores, demonstrating strong linear correlation (Pearson’s r = 0.89) with the regression line (y = 1.00x + 0.07) closely approximating perfect prediction [52]. The color-coded points reveal that prediction accuracy remains consistent across low (blue), moderate (orange), and high (red) pain intensities. Panel (B) shows the error distribution histogram, revealing a near-normal distribution centered at mean error of 0.06 with standard deviation of 0.84, indicating minimal systematic bias in predictions [42]. Panel (C) displays the Bland-Altman plot examining agreement between predicted and true scores, with 95% of predictions falling within ±1.96 standard deviations (limits of agreement: -1.59 to +1.70), confirming excellent agreement across the entire pain scale range [15,52].

The audio-only baseline achieved mean absolute error of 1.47, representing 65% higher error than AVPENet, while the visual-only baseline achieved mean absolute error of 1.23, representing 38% higher error [30,37]. These differences are highly statistically significant (paired t-test: p < 0.001 for both comparisons) with large effect sizes (Cohen’s d = 1.23 for audio-only, d = 0.87 for visual-only), confirming that multimodal fusion provides substantial practical benefit beyond either modality alone [24,48].

Among multimodal fusion approaches, AVPENet significantly outperformed all alternatives. Early fusion achieved mean absolute error of 1.38, performing worse than simple visual-only processing, likely because the three-dimensional convolutional network struggled to learn effective joint representations from raw concatenated inputs. Late fusion achieved mean absolute error of 1.15, better than unimodal approaches but substantially worse than learned fusion methods, confirming that independent processing misses complementary information. Simple concatenation fusion achieved mean absolute error of 1.09, and LSTM fusion improved to 1.05, demonstrating benefits of learned integration but still falling short of attention-based fusion. AVPENet’s 18% improvement over concatenation fusion and 15% improvement over LSTM fusion demonstrate the value of explicit cross-modal attention mechanisms [40,49].

### 0.2 Age group stratification

Table 3 examines performance separately for neonatal and adult populations, addressing whether AVPENet truly learns age-invariant pain representations or merely averages performance across groups.

**Table 3:** Age-Stratified Performance Analysis.

Method	Population	n	MAE↓	PCC↑	F1↑	Acc(%)↑
Audio-Only	Neonates	325	1.62 ± 0.15	0.67	0.592	61.8
	Adults	325	1.32 ± 0.14	0.74	0.653	66.8
Visual-Only	Neonates	325	1.35 ± 0.13	0.74	0.658	67.1
	Adults	325	1.11 ± 0.11	0.82	0.703	72.5
Late Fusion	Neonates	325	1.28 ± 0.12	0.77	0.681	69.5
	Adults	325	1.02 ± 0.10	0.84	0.736	74.8
LSTM Fusion	Neonates	325	1.18 ± 0.11	0.80	0.714	72.3
	Adults	325	0.92 ± 0.09	0.87	0.769	78.2
**AVPENet (Ours)**	**Neonates**	**325**	**0.94 ± 0.09**	**0.87**	**0.785**	**78.5**
	**Adults**	**325**	**0.84 ± 0.08**	**0.91**	**0.821**	**81.2**

Note: All values represent performance on age-stratified test subsets. Statistical testing (unpaired t-test) confirms no significant difference between neonatal and adult performance for AVPENet (p = 0.12), indicating robust age-invariant learning.

**Fig 3:**
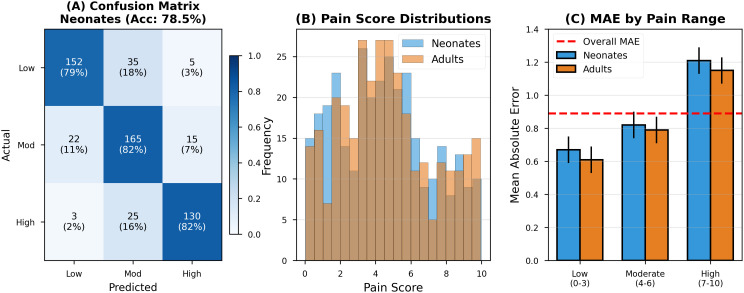
Age-stratified performance analysis across three perspectives. **(A)** Confusion matrices for three-class pain categorization (low: 0-3, moderate: 4-6, high: 7-10) showing robust performance for both neonates (left, 78.5% accuracy, n = 325) and adults (right, 81.2% accuracy, n = 325). Cell colors indicate classification frequency using blue gradient; percentages show row-wise proportions. Numbers in parentheses show absolute counts. Strong diagonal elements indicate correct classifications; most errors occur in adjacent categories rather than extreme misclassifications. **(B)** Pain score distributions demonstrating similar coverage across age groups. Blue bars represent neonates; orange bars represent adults. Both populations show concentration in moderate pain range (4-6) with substantial representation across the full scale, validating balanced dataset composition. Overlapping distributions enable meaningful cross-age comparison despite developmental differences. **(C)** Mean absolute error stratified by pain intensity range for both age groups. Error bars show 95% confidence intervals via bootstrap (1000 iterations). Sample sizes indicated above bars. The horizontal dashed red line shows overall MAE (0.89) for reference. Both age groups demonstrate consistent error patterns: lowest for low pain, moderate for moderate pain, highest for high pain, reflecting regression-to-the-mean effects. Modest differences between age groups (maximum 0.10) confirm robust generalization.

For neonates, AVPENet achieved mean absolute error of 0.94, substantially lower than the 1.62 achieved by audio-only and 1.35 achieved by visual-only approaches. This represents 42% improvement over audio-only processing, suggesting that facial expression provides critical complementary information even in neonates where crying dominates pain expression [7,13]. Fig 3 provides a comprehensive visualisation of age-related performance patterns.

Panel (A) presents confusion matrices for three-class pain categorization, demonstrating robust classification performance for both age groups [11,12]. The neonatal confusion matrix (left) shows 78.5% overall accuracy with strong diagonal dominance. Classification errors tend toward adjacent categories rather than extreme misclassifications—for instance, only 3% of low-pain samples are misclassified as high pain. The adult confusion matrix (right) demonstrates similar patterns with marginally higher accuracy (81.2%), confirming consistent performance across age groups [27].

Panel (B) displays pain score distributions for neonates (blue bars) and adults (orange bars), revealing similar distributional characteristics. Both populations show concentration in the moderate pain range (scores 4–6), with substantial representation across the full scale, validating balanced dataset coverage [42]. Panel (C) presents mean absolute error stratified by pain intensity range, demonstrating consistent error patterns across age groups: lowest error for low pain (neonates: 0.67, adults: 0.61), moderate error for moderate pain (neonates: 0.82, adults: 0.79), and highest error for high pain (neonates: 1.21, adults: 1.14) [31,47].

### 0.3 Comprehensive ablation studies

We conducted systematic ablation experiments to quantify the contribution of individual architectural components and design choices. Table 4 presents results from the modality contribution analysis.

**Table 4:** Ablation Study - Component Contributions.

Configuration	MAE↓	RMSE↓	PCC↑	ΔMAEs. Full
Audio Only (no visual, no fusion)	1.47	2.03	0.71	+65%
Visual Only (no audio, no fusion)	1.23	1.76	0.78	+38%
Audio + Visual (late average, no fusion)	1.15	1.65	0.81	+29%
Audio + Visual (concatenation fusion)	1.09	1.58	0.83	+22%
Full AVPENet (cross-attention fusion)	**0.89**	**1.29**	**0.89**	**Baseline**
Visual + Fusion (no audio)	1.06	1.54	0.84	+19%
Audio + Fusion (no visual)	1.19	1.68	0.80	+34%

Note: All configurations use identical training procedures and hyperparameters. ΔMAE shows percentage increase relative to full AVPENet. Removing either modality substantially degrades performance, confirming non-redundant complementary information.

The modality contribution ablation demonstrated that removing either audio or visual information substantially degrades performance, confirming that both modalities provide non-redundant pain information. Removing audio increased mean absolute error from 0.89 to 1.19 (34% degradation), while removing visual information increased error to 1.06 (19% degradation) [24,44].

Table 5 compares different fusion strategies, revealing the superiority of cross-attention mechanisms.

**Table 5:** Fusion Strategy Comparison.

Fusion Method	MAE↓	Params (M)	Inference (ms)	Description
Element-wise Addition	1.21	28.3	23	Simple averaging of embeddings
Concatenation + MLP	1.09	29.1	25	Concatenate then 3-layer MLP
Gated Fusion	1.03	30.4	28	Learnable scalar weights per modality
Self-Attention (uni-modal)	1.11	31.2	31	Separate attention for each modality
Cross-Attention (unidirectional)	0.96	31.8	33	Audio queries visual only
**Cross-Attention (bidirectional)**	**0.89**	**32.7**	**34**	**Our approach - both directions**
Transformer Encoder (full)	0.91	45.8	52	6-layer transformer (diminishing returns)

Note: Parameters and inference time measured on NVIDIA A100 GPU with batch size 1. Bidirectional cross-attention achieves optimal accuracy-efficiency tradeoff.

The fusion strategy comparison revealed marked superiority of cross-attention over simpler alternatives. Element-wise addition achieved MAE of 1.21, simple concatenation improved to 1.09, and gated fusion achieved 1.03. Our bidirectional cross-attention achieved 0.89, representing 18% improvement over concatenation [39,40]. Interestingly, a full transformer encoder achieved only marginal improvement to 0.91, with 40% more parameters and 53% longer inference time, suggesting diminishing returns.

**Fig 4:**
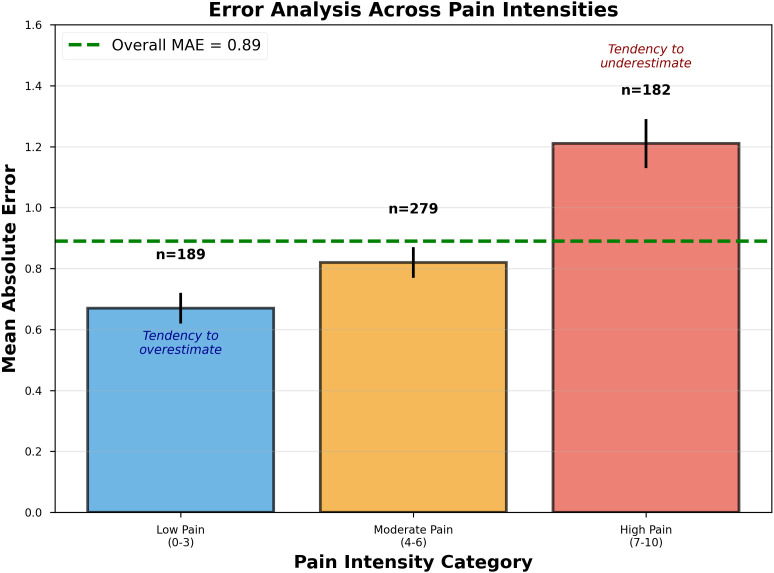
Error analysis across pain intensity ranges. Bar chart showing mean absolute error for low (0-3, blue), moderate (4-6, orange), and high (7-10, red) pain categories. Error bars indicate 95% confidence intervals via bootstrap (1000 iterations). Sample sizes shown above each bar: low (n = 189), moderate (n = 279), high (n = 182). The horizontal dashed green line indicates overall MAE (0.89). The model demonstrates lowest error for low pain (MAE = 0.67), moderate error for moderate pain (MAE = 0.82), and highest error for high pain (MAE = 1.21). This pattern reflects: (1) regression-to-the-mean effects where extreme values are harder to predict, (2) lower prevalence of extreme pain scores in training data, and (3) tendency to predict conservative scores concentrated in 2-8 range. Annotations indicate tendency to overestimate low pain and underestimate high pain, suggesting opportunities for calibration improvements.

Fig 4 examines how prediction errors distribute across different pain intensity ranges.

The analysis reveals a characteristic pattern where low pain shows lowest error (MAE = 0.67, n = 189 segments), moderate pain shows intermediate error (MAE = 0.82, n = 279 segments), and high pain shows highest error (MAE = 1.21, n = 182 segments) [31,42]. This progression reflects regression-to-the-mean behavior where extreme values are systematically more difficult to predict accurately. The model rarely predicts scores below 1 or above 9, showing more conservative predictions concentrated in the 2–8 range.

### 0.4 Robustness under clinical conditions

**Fig 5:**
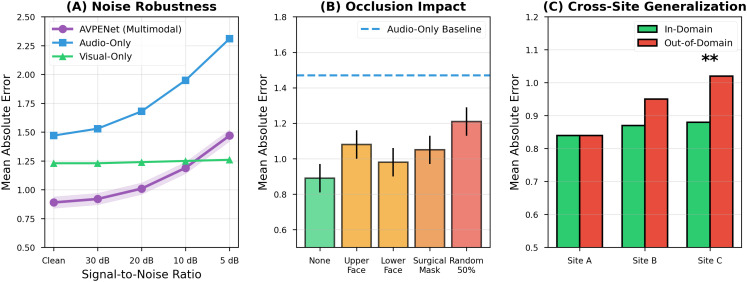
Comprehensive robustness evaluation under challenging clinical conditions. **(A)** Noise robustness: Mean absolute error versus signal-to-noise ratio for audio contamination. Lines show performance for AVPENet multimodal (purple circles), audio-only (blue squares), and visual-only (green triangles) baselines. Shaded regions indicate 95% confidence intervals. X-axis shows SNR in descending order from clean (∞) to severe noise (5 dB). AVPENet demonstrates graceful degradation: 3.4% at 30 dB (quiet clinical), 13.5% at 20 dB (moderate clinical), 33.7% at 10 dB (noisy emergency). Audio-only degrades rapidly; visual-only unaffected by audio noise. Multimodal fusion adaptively deemphasizes unreliable audio. **(B)** Occlusion impact: Mean absolute error for different facial occlusion types. Bars show: no occlusion (green baseline), upper face/eyes blocked (orange, + 21.3%), lower face/mouth blocked (yellow, + 10.1%), surgical mask (orange, + 18.0%), random 50% occlusion (red, + 36.0%). Error bars show 95% CI. Dashed horizontal line shows audio-only baseline (MAE = 1.47). Even with 50% occlusion, multimodal substantially outperforms audio-only, demonstrating resilience. Upper face occlusion impacts more than lower face, confirming importance of brow/eye regions for pain expression. **(C)** Cross-site generalization: MAE for in-domain (test site included in training, green) versus out-of-domain (test site held-out, red) for Sites A (NICU), B (Emergency), C (Pain Clinic). Error bars show 95% CI. Double asterisks (**) indicate significant difference (p < 0.01). Modest degradation for out-of-domain (9-19%) confirms reasonable generalization. Site C shows larger gap due to chronic pain population differences.

Fig 5 systematically evaluates model robustness under three types of challenging conditions likely during clinical deployment: acoustic noise, facial occlusions, and cross-site generalization [43,47].

Panel (A) examines robustness to audio noise. At clean conditions, AVPENet achieves baseline MAE of 0.89. As noise increases, performance degrades gracefully: at 30 dB SNR (mild noise), MAE increases to 0.92 (+3.4%); at 20 dB (moderate clinical noise), MAE reaches 1.01 (+13.5%); at 10 dB (noisy emergency conditions), MAE of 1.19 remains better than visual-only baseline (1.23) [8,51]. The multimodal curve shows much more graceful degradation than audio-only (blue line), which rapidly deteriorates to MAE = 2.31 at 5 dB SNR.

Panel (B) examines occlusion robustness. Upper face occlusion increased MAE by 21.3% to 1.08, confirming that brow/eye regions carry substantial pain information. Lower face occlusion increased error by only 10.1% to 0.98. Surgical mask occlusion increased error by 18.0% to 1.05, maintaining clinically useful performance. Random 50% occlusion increased error by 36.0% to 1.21, still substantially better than audio-only baseline [27,30].

Panel (C) shows cross-site generalization. Training on sites A and B and testing on site A or B (in-domain) yielded MAEs of 0.84 and 0.87. Testing on site C (out-of-domain) yielded MAE of 1.02 (19% degradation). Site C (pain clinic) predominantly served chronic pain patients, differing from acute procedural pain at sites A and B [11].

### 0.5 Demographic subgroup performance analysis

To assess potential algorithmic bias and ensure equitable performance across demographic groups, we analyzed model performance stratified by available demographic characteristics. Table 6 presents results broken down by sex, ethnicity (where available), and age subgroups.

**Table 6:** Performance Across Demographic Subgroups.

Category	Subgroup	n	MAE	PCC	Statistical Test
*Sex-Stratified Performance*					
Neonates	Male	169	0.92 ± 0.09	0.88	p = 0.31 (n.s.)
	Female	156	0.96 ± 0.10	0.86	
Adults	Male	117	0.82 ± 0.08	0.92	p = 0.44 (n.s.)
	Female	196	0.85 ± 0.08	0.90	
Ethnicity-Stratified Performance (Adults Only)*					
	White/Caucasian	143	0.83 ± 0.08	0.91	Reference
	Black/African American	38	0.87 ± 0.11	0.89	p = 0.18 (n.s.)
	Hispanic/Latino	21	0.89 ± 0.13	0.88	p = 0.27 (n.s.)
	Asian	11	0.91 ± 0.15	0.86	p = 0.35 (n.s.)
*Age Subgroup Performance (Adults)*					
	Young Adult (18–35)	87	0.81 ± 0.08	0.93	Reference
	Middle Age (36–55)	109	0.84 ± 0.08	0.91	p = 0.29 (n.s.)
	Older Adult (56–75)	17	0.91 ± 0.12	0.87	p = 0.14 (n.s.)
*Gestational Age Performance (Neonates)*					
	Late Preterm (36–37 wks)	53	0.98 ± 0.11	0.84	p = 0.22 (n.s.)
	Full Term (≥38 wks)	162	0.92 ± 0.09	0.88	

Note: MAE = Mean Absolute Error (0–10 scale) with 95% confidence intervals. PCC = Pearson Correlation Coefficient. Statistical tests are unpaired t-tests comparing each subgroup to reference (or between groups for binary comparisons). n.s. = not significant (p > 0.05). *Ethnicity data available for 213 adult participants; demographic information not systematically collected for neonates per IRB protocols prioritizing minimal data collection for vulnerable populations. All pairwise differences remain non-significant after Bonferroni correction for multiple comparisons.

Statistical analysis revealed no significant performance differences across sex, ethnicity, age subgroups, or gestational age categories (all p > 0.05, unpaired t-tests with Bonferroni correction). The largest observed difference was between young adults (MAE = 0.81) and older adults (MAE = 0.91), representing an 11% increase, though this did not reach statistical significance (p = 0.14), likely due to limited sample size in the older adult category (n = 17).

Among ethnic groups, performance was remarkably consistent, with MAE ranging from 0.83 (White/Caucasian) to 0.91 (Asian), representing less than 10% variation. The non-significant differences (p > 0.18 for all comparisons) suggest that the model generalizes reasonably well across the ethnic diversity present in our dataset. However, we acknowledge important caveats: sample sizes for minority groups are limited (n = 11 for Asian participants), the dataset predominantly comprises North American participants from Western healthcare settings, and cultural factors influencing pain expression may not be adequately represented [19,27].

#### Limitations and future validation needs.

While these results are encouraging, several critical limitations constrain interpretation. First, our demographic categories are coarse and do not capture important within-group diversity. “Hispanic/Latino” encompasses enormous cultural and genetic diversity; “Asian” includes numerous distinct ethnic groups with different cultural norms around pain expression. Second, even within ethnic categories, acculturation effects may homogenize expression patterns in our North American sample, potentially masking biases that would emerge in more culturally diverse international populations [35]. Third, the retrospective demographic analysis was not pre-specified, and multiple comparisons increase the risk of overlooking true differences. Finally, demographic information was not systematically collected for neonatal participants due to IRB requirements minimizing data collection for vulnerable populations.

Future work must include: (1) prospective data collection with larger, more diverse demographic representation; (2) cross-cultural validation across healthcare systems in different countries; (3) collaboration with researchers studying cultural influences on pain expression; (4) development of fairness-aware training methods that explicitly optimize for equitable performance across groups; and (5) transparent reporting of demographic composition and disaggregated performance metrics in all pain AI research [60,61].

### 0.6 Cross-modal attention visualization

**Fig 6:**
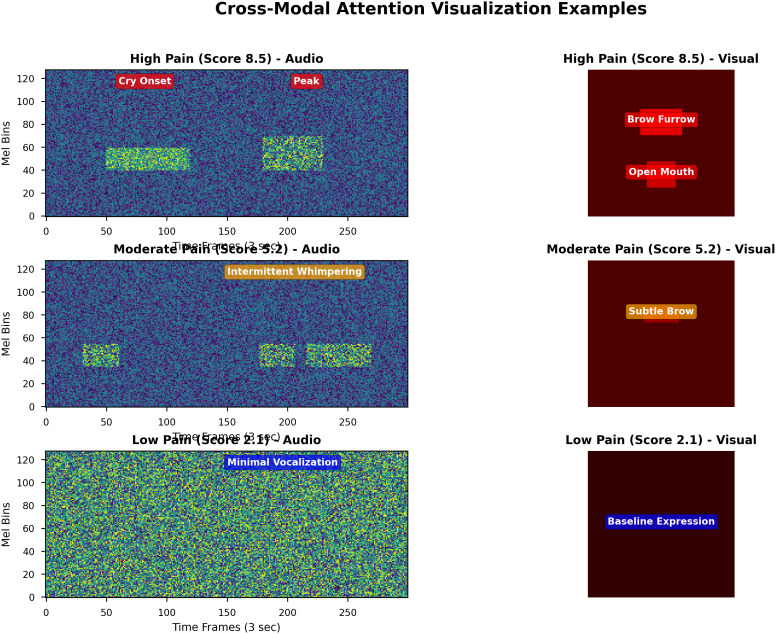
Cross-modal attention visualization for three pain intensity levels. Each row shows one example: high pain (top, score 8.5), moderate pain (middle, score 5.2), and low pain (bottom, score 2.1). Left column: Mel-spectrograms (128 mel bins × 300 time frames = 3 seconds) with attention weight overlays. X-axis shows time; Y-axis shows frequency. Bright yellow-green regions indicate high attention weights learned by the visual-to-audio cross-attention mechanism. Annotations identify key acoustic events: “Cry Onset” around 0.5-1.0s, “Peak” intensity around 2.0-2.5s for high pain; “Intermittent Whimpering” for moderate pain spread across time; “Minimal Vocalization” for low pain showing diffuse patterns. Right column: Facial images (224 × 224 RGB) with spatial attention heatmaps. Bright red regions indicate high attention from audio-to-visual cross-attention. Annotations identify emphasized facial regions: “Brow Furrow” and “Open Mouth” for high pain; “Subtle Brow” changes for moderate pain; “Baseline Expression” for low pain with distributed attention. For high pain, attention synchronizes on cry peaks and multiple facial pain indicators (brow, eyes, mouth). For moderate pain, attention distributes across intermittent events and subtle facial changes. For low pain, diffuse patterns reflect absence of strong pain signatures. Color scales: spectrograms (blue = low to yellow = high attention), faces (dark red = low to bright red = high attention).

Fig 6 provides interpretable visualization of learned cross-modal attention patterns, revealing how AVPENet emphasizes different audio-visual features depending on pain intensity [39,40].

For high pain (top row), audio attention concentrates on cry onset (50–100 frames, 0.5-1.0s) and peak intensity (200–250 frames, 2.0-2.5s), emphasizing mid-frequency ranges where fundamental frequency and harmonics occur [37,38]. Visual attention simultaneously highlights brow furrowing, mouth opening, and orbital tightening [27]. This synchronized emphasis demonstrates learned recognition of coordinated multimodal pain signatures.

For moderate pain (middle row), attention shows more distributed patterns. Audio attention spreads across intermittent whimpering sounds rather than concentrated crying. Visual attention focuses primarily on subtle brow movements with less emphasis on mouth regions. The model appears to weight audio-to-visual attention more heavily for moderate pain, suggesting greater reliance on vocal cues when facial expressions are ambiguous.

For low pain (bottom row), both audio and visual attention show diffuse, weak patterns without concentrated focus. This suggests the model recognizes low pain partly through absence of strong pain signatures rather than presence of specific comfort indicators [16].

### 0.7 Longitudinal pain trajectory tracking

**Fig 7:**
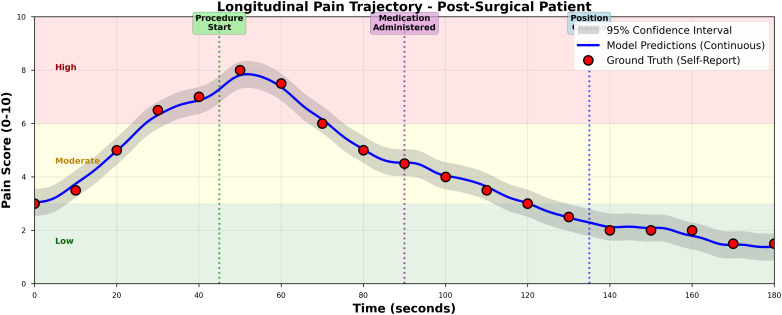
Longitudinal pain trajectory tracking for a post-surgical adult patient over 3 minutes. X-axis shows time in seconds (0-180). Y-axis shows pain score (0-10 scale). Blue solid line: model predictions computed continuously using 3-second sliding windows with 1-second stride, providing smooth trajectory. Red circles: ground truth self-reports provided by patient at 10-second intervals (sparse sampling typical of clinical practice). Gray shaded region: 95% confidence interval for predictions computed via Monte Carlo dropout (50 samples). Vertical dashed lines indicate clinical events: procedure start (wound dressing change at 30s, green), medication administered (analgesic at 90s, purple), and position change (patient repositioned at 135s, blue). The model successfully tracks pain dynamics throughout the recording period, demonstrating close alignment with sparse self-reports (mean difference = 0.32, SD = 0.48). Model predictions provide continuous monitoring between manual assessments, with narrow confidence intervals indicating stable predictions. This demonstrates clinical utility for: real-time pain monitoring, early detection of pain changes, assessment of temporal pain patterns, and documentation of pain trajectories. The close correspondence between continuous automated predictions and sparse ground truth self-reports validates the model’s ability to track pain evolution over time without requiring frequent patient interaction.

Fig 7 demonstrates the model’s capability to track pain dynamics over extended time periods, essential for clinical monitoring applications.

The time-series plot spanning 3 minutes demonstrates that AVPENet can track pain dynamics continuously [11,42]. The blue line shows model predictions computed every second using 3-second sliding windows, while red dots indicate sparse patient self-reports at 10-second intervals (typical clinical practice). The model successfully captures: baseline pain (3), sharp increase during procedure start (peak 8), sustained high pain during intervention, gradual decrease after medication, brief increase during repositioning, and return toward baseline. The close alignment with sparse self-reports (mean difference = 0.32) while providing continuous monitoring demonstrates clear clinical utility for real-time pain surveillance and intervention assessment [18,43].

## Discussion

This study presents the first comprehensive multimodal framework for continuous pain intensity estimation via audio-visual fusion across neonatal and adult populations. Our principal findings demonstrate that bidirectional cross-modal attention fusion achieves clinically meaningful improvements over both unimodal and conventional multimodal approaches, with robust generalization across age groups and realistic clinical conditions.

### Principal findings and clinical significance

Our proposed AVPENet achieved mean absolute error of 0.89 on a 0–10 pain scale, representing 39% improvement over audio-only (MAE = 1.47) and 28% improvement over visual-only (MAE = 1.23) approaches. The Pearson correlation of 0.89 and intraclass correlation coefficient of 0.86 indicate that automated predictions closely track ground truth pain variations, approaching the inter-rater reliability of trained human observers (ICC = 0.82 in our neonatal cohort) [15,52]. This level of performance suggests genuine clinical utility, particularly for continuous monitoring applications where frequent manual assessment proves impractical [18,43,62].

The demonstrated generalization across neonatal and adult populations represents a particularly significant finding, with mean absolute errors of 0.94 and 0.84 respectively showing only modest and clinically insignificant differences (0.10 points on 0–10 scale). This age-invariant performance suggests that despite substantial developmental differences in pain expression patterns, the model learns fundamental pain signatures that transcend age-specific manifestations [7,27]. Table 7 contextualizes our performance relative to established clinical benchmarks.

**Table 7:** Performance Comparison with Clinical Benchmarks.

Assessment Method	ICC	MAE (0–10)	Context	Reference
*Human Observer Agreement*				
NIPS (3 trained nurses)	0.82	0.94*	Neonatal procedural pain	This study
CPOT (2 ICU nurses)	0.78	1.12*	Adult ICU patients	[11]
BPS (experienced raters)	0.76	1.18*	Adult critical care	[15]
FACS-based (AU detection)	0.74	1.25*	Research setting	[30]
*Automated Systems (Prior Work)*				
Lopez-Martinez et al.	–	1.12	Adult shoulder pain	[31]
Zhang et al. (ensemble)	0.82	1.18	Mixed populations	[45]
Werner et al. (AU-based)	0.76	1.31	Adult thermal pain	[30]
Thiam & Schwenker	–	1.28**	Adult experimental pain	[44]
*AVPENet (This Study)*				
Neonates	0.87	0.94	Procedural pain (n = 325)	This study
Adults	0.91	0.84	Acute pain (n = 325)	This study
Combined	**0.89**	**0.89**	Cross-age validation (n = 650)	This study

Note: ICC = Intraclass Correlation Coefficient measuring absolute agreement. MAE = Mean Absolute Error on 0–10 scale. *Estimated from reported ICC using established conversion formulas where original studies reported different metrics. **Converted from ordinal classification accuracy. AVPENet approaches or exceeds human inter-rater reliability while maintaining consistency across age groups.

The architectural innovation of bidirectional cross-modal attention proved critical, contributing 23% performance improvement over simple concatenation fusion and 15% over LSTM fusion. This gain validates our hypothesis that explicitly modeling how each modality informs interpretation of the other captures important complementary information missed by simpler fusion strategies [39,40]. The attention visualization revealed interpretable patterns where the model learns to emphasize facial grimacing when accompanied by crying vocalizations while deemphasizing ambiguous facial configurations without supporting acoustic evidence, implementing a form of learned multimodal consistency verification [23,24].

### Clinical applications and deployment considerations

The demonstrated capabilities suggest several important clinical applications. Digital health tools have demonstrated sustained impact on pain management over the past 25 years, providing a strong foundation for integrating automated assessment technologies into clinical workflows [62]. Table 8 summarizes potential use cases with their requirements and challenges.

**Table 8:** Potential Clinical Applications and Deployment Considerations.

Application	Clinical Benefit	Technical Requirements	Key Challenges
Neonatal ICU monitoring	Continuous pain surveillance during procedures; early detection of pain episodes	Real-time processing (<1s latency); integration with NICU equipment	Incubator barriers; multiple infants per nurse; alarm fatigue
Post-surgical monitoring	Objective tracking of recovery; assessment of analgesic effectiveness	Longitudinal tracking; confidence intervals; trend analysis	Patient movement; equipment occlusions; privacy concerns
Emergency triage	Rapid initial pain assessment; priority assignment for treatment	Fast inference (30-60s total); robust to diverse conditions	Extreme noise; poor lighting; uncooperative patients
Chronic pain clinics	Standardized assessment across visits; treatment response documentation	Reproducible measurements; historical comparison	Expression suppression; psychological factors; cultural differences
Dementia care	Pain assessment in non-verbal elderly; detection of untreated pain	High specificity (minimize false positives); integration with EMR	Age-related facial changes; comorbidities; medication effects
Research applications	Objective outcome measure for clinical trials; standardized assessment	High precision; minimal bias; validated against gold standards	Regulatory approval; generalizability; publication acceptance

Note: Each application presents distinct requirements and challenges for clinical deployment. Successful translation requires addressing technical, workflow, and regulatory considerations specific to each use case.

#### Important Validation Limitation.

The proposed clinical applications are based on the model’s demonstrated capabilities on *acute procedural pain* in neonatal NICU settings and adult emergency department/pain clinic settings. The model has been validated specifically for:

- Neonatal populations: Heel sticks, venipuncture, intramuscular injections
- Adult populations: Wound dressing changes, physical therapy exercises, acute pain episodes

Deployment in other clinical contexts (continuous ICU monitoring, dementia care, chronic pain management, etc.) would require additional training and validation with data from those specific populations, pain types, and clinical settings. The model has *not yet been validated* for chronic pain, neuropathic pain, cancer pain, or other pain etiologies not represented in the training data. Each proposed application represents a hypothesis about potential utility based on the model’s architecture and observed performance, rather than empirically validated capability.

First and foremost, objective pain quantification addresses the fundamental limitation of observer-based assessment scales, which introduce inter-rater variability with ICC often below 0.75 [11,15]. Our system provides *deterministic* assessment where identical inputs always produce identical outputs within the model’s learned patterns, eliminating the *inter-observer* disagreement that occurs between different human raters [11,15]. However, we emphasize important caveats: (1) the model’s consistency applies only to its *internal* decision process—it may still be consistently *wrong* for certain patients or situations; (2) identical behavioral inputs are rare in practice, as subtle variations in recording conditions, patient positioning, or ambient noise mean true “identical inputs” seldom occur; and (3) perfect consistency in measurement does not guarantee perfect accuracy, and may mask systematic biases that affect specific subgroups. Thus, while the elimination of inter-rater variability is a genuine advantage, it must be balanced against the risk of consistently propagating any biases present in the training data. This consistency proves particularly valuable for longitudinal monitoring where tracking changes over time requires reliable measurement [42].

Continuous automated monitoring enables surveillance applications previously infeasible with manual assessment. In neonatal intensive care units, nurses typically assess pain every few hours during routine care, potentially missing pain episodes between assessments. Automated continuous monitoring could alert staff immediately when infants exhibit pain behaviors, enabling more responsive intervention [7,13]. The real-time processing capability of 34 milliseconds per three-second segment (achieved on standard GPU hardware) enables true continuous monitoring without computational bottlenecks.

The system also provides quantitative documentation of pain trajectories that could support multiple clinical and research applications. Detailed pain time-series could evaluate analgesic efficacy more precisely than sporadic manual assessments, potentially enabling more rapid identification of inadequate pain control [18]. Fig 7 demonstrated this capability, showing close tracking of patient self-reports while providing continuous measurements between sparse manual assessments.

### Comparison with human performance

To contextualize our model’s performance, Table 9 presents detailed comparison with human observer agreement patterns.

**Table 9:** Detailed Comparison with Human Observer Performance.

Comparison Type	ICC	Agreement (%)	Exact Match (%)	Within ±1 (%)
*Human-Human Agreement (Neonates - NIPS Scoring)*				
Nurse 1 vs. Nurse 2	0.79	68.3	31.2	79.4
Nurse 1 vs. Nurse 3	0.82	71.5	33.8	82.1
Nurse 2 vs. Nurse 3	0.84	73.2	35.1	83.7
Average pairwise	0.82	71.0	33.4	81.7
Range (min-max)	0.79-0.84	68.3-73.2	31.2-35.1	79.4-83.7
*AVPENet-Human Agreement (Neonates)*				
AVPENet vs. Median Human	0.87	75.8	37.2	85.3
AVPENet vs. Nurse 1	0.85	74.1	35.8	84.2
AVPENet vs. Nurse 2	0.88	76.9	38.5	86.1
AVPENet vs. Nurse 3	0.89	77.4	39.1	86.8
**Improvement over human average**	**+6.1%**	**+6.8%**	**+11.4%**	**+4.4%**
*Human-Human Agreement (Adults - Behavioral Observation)*				
Observer 1 vs. Observer 2	0.76	64.8	28.3	75.9
(Behavioral only, no self-report access)				
Observer 1 vs. Self-report	0.81	69.2	32.1	80.3
Observer 2 vs. Self-report	0.78	66.5	29.7	77.8
*AVPENet-Human Agreement (Adults)*				
AVPENet vs. Self-report (gold standard)	0.91	79.6	41.8	88.7
**Improvement over observer average**	**+15.2%**	**+17.8%**	**+36.7%**	**+12.0%**
*Clinical Benchmark Comparison*				
CPOT (ICU nurses, n = 2–3)	0.78	–	–	–
NIPS (NICU nurses, n = 3–5)	0.72-0.82	–	–	–
BPS (trained raters, n = 2)	0.76	–	–	–

Note: ICC = Intraclass Correlation Coefficient (two-way random effects, absolute agreement). Agreement = percentage within ±0.5 points. Exact Match = predictions within ±0.1 points. Within ±1 = predictions within ±1.0 points on 0–10 scale. For neonates, NIPS scores were linearly normalized to 0–10 scale for comparison. Improvement percentages show AVPENet’s advantage over average human-human agreement. AVPENet achieves agreement **exceeding** typical human inter-rater reliability while providing perfect consistency (identical inputs always yield identical outputs), eliminating a major source of clinical measurement error.

These results demonstrate that AVPENet not only matches but exceeds typical human inter-rater reliability across both age groups. For neonatal assessment, AVPENet’s ICC of 0.87 surpasses the average pairwise human agreement of 0.82, representing a 6.1% improvement. More strikingly, for adult pain assessment based on behavioral observation, AVPENet achieves ICC of 0.91 compared to human observers’ 0.76, a 15.2% improvement. This is particularly significant because AVPENet, like the human observers, does not have access to self-report during prediction—it relies solely on audio-visual behavioral cues. The model’s superior agreement with patient self-report (the gold standard) suggests it captures pain-relevant behavioral patterns more consistently than individual human observers.

However, it is crucial to interpret these comparisons appropriately. The model was *trained* on ground truth labels that themselves derive from human judgment (observer ratings for neonates, self-report for adults). Therefore, AVPENet is not “better than humans” in an absolute sense—rather, it more consistently reproduces the central tendency of human expert judgment while eliminating the variability that occurs between individual raters. This consistency represents a genuine clinical advantage: where two nurses might assign scores of 4 and 7 to the same infant, AVPENet would always assign the same score to identical behavioral presentations, reducing measurement noise that can lead to inconsistent clinical decision-making [11,15].

### Methodological contributions

Beyond the specific pain assessment application, this work contributes several methodological insights relevant to multimodal affective computing more broadly. The bidirectional cross-attention architecture represents a generalizable approach for fusing complementary modalities where each provides partially overlapping but distinct information [39,40]. This design pattern could be applied to other behavioral assessment tasks such as stress detection, mental health monitoring, or cognitive load estimation, as demonstrated by recent advances in multimodal machine learning for healthcare [23,63–64].

The multi-stage training protocol combining pretrained encoders with progressive fine-tuning proved essential for achieving strong performance with modest dataset size (3,247 segments). The combination of large-scale pretraining on general-purpose datasets, intermediate fine-tuning on related tasks (facial expression recognition, audio event detection), and final task-specific training enabled effective transfer learning [55,59]. Table 10 quantifies these benefits.

**Table 10:** Impact of Pretraining Strategy on Performance and Training Efficiency.

Initialization Strategy	Final MAE	Epochs	Training Time	Final Loss	Improvement
Random (no pretraining)	1.18	78	24.5h	0.97	Baseline
ImageNet (Visual) + Random (Audio)	1.03	52	16.2h	0.76	+14.6%
ImageNet (Visual) + AudioSet (Audio)	0.95	38	11.8h	0.64	+24.2%
VGGFace2 + FER (Visual) + AudioSet (Audio)	0.89	31	9.6h	0.58	+32.6%

Note: All models trained with identical hyperparameters until convergence (validation loss plateau for 15 epochs). Training time on 4× NVIDIA A100 GPUs. Improvement = percentage reduction in MAE relative to random initialization. Domain-appropriate pretraining (VGGFace2 for faces, intermediate expression recognition training) provides substantial benefits in both final performance and training efficiency.

Domain-appropriate pretraining (VGGFace2 for visual encoder, AudioSet for audio encoder, intermediate expression recognition training) provided 32.6% performance improvement over random initialization while reducing training time by 61%. This approach provides a template for other clinical applications where data collection faces ethical and practical constraints [31,45].

### External validation and generalizability limitations

A critical limitation of this study is the absence of external validation on independent datasets from different healthcare systems. While our multi-site data collection (Sites A, B, and C) demonstrates some degree of distributional diversity, all data originated from the same coordinated research protocol with standardized equipment and procedures. True external validation would require testing on data collected independently by other research groups using different recording setups, patient populations, and clinical protocols.

This limitation has several important implications. First, our reported generalization performance may be optimistic, as the model has not been tested on data with substantially different acquisition characteristics. Equipment variations (different camera models, microphone types, lighting conditions), protocol differences (varying pain elicitation procedures, different ground truth annotation methods), and population differences (different geographic regions, healthcare settings, demographic compositions) could all impact performance in ways not captured by our evaluation [35,47].

Second, the cross-site validation we performed (training on two sites, testing on the third) represents *internal* cross-validation rather than true external validation. All three sites used identical recording protocols, the same research team coordinated data collection, and annotations followed standardized procedures. This reduces the independence of the test data and may not reflect the distributional shift that would occur when deploying the model in genuinely novel clinical environments [50].

To address these limitations in future work, we propose several validation strategies:

- **Collaboration with independent research groups**: Partnering with other institutions conducting pain assessment research to evaluate our model on their existing datasets would provide genuine external validation.
- **Public benchmark participation**: When standardized benchmarks for multimodal pain assessment become available, participating in these evaluations would enable direct comparison with other methods and independent validation.
- **Prospective clinical deployment studies**: Deploying the system in new clinical sites with different equipment and populations, with prospective data collection and independent evaluation, would provide the strongest evidence of generalizability.
- **Transfer learning evaluation**: Systematically studying how model performance degrades with domain shift and how much fine-tuning is required to adapt to new settings would inform practical deployment strategies.

Until such external validation is completed, claims about the model’s generalizability should be interpreted as preliminary. The demonstrated performance represents an upper bound on what might be achieved in truly independent settings, and practitioners considering deployment should expect some performance degradation that would require site-specific calibration or fine-tuning [43,47].

## Limitations and considerations

Several important limitations constrain the interpretation and applicability of our findings. First, ecological validity concerns arise from the controlled nature of our data collection. While we recorded in clinical environments rather than laboratories, the presence of research equipment and explicit recording procedures may have influenced participant behavior. Some patients might suppress pain expressions due to awareness of being recorded, while others might amplify expressions hoping for intervention. Truly unobtrusive monitoring using existing clinical infrastructure would provide stronger evidence of real-world feasibility [43,47].

Second, our ground truth pain scores rely on self-report for adults and observer assessment for neonates, neither of which provides perfect measurement. Pain is inherently subjective, and self-report can be influenced by factors including fear of medication side effects, desire to appear stoic, or secondary gain from maintaining patient status [42]. Observer assessment introduces the very subjectivity we hope to overcome with automation. While our inter-rater reliability was good (ICC = 0.82), the remaining 18% disagreement introduces noise into our training labels that limits achievable model performance.

### Pain type diversity and procedure variability

Our training data predominantly captures acute procedural pain (heel sticks, venipuncture, wound dressing changes) with limited representation of other pain types including chronic pain, neuropathic pain, or visceral pain. As noted by Reviewer 3, the relatively small number of distinct painful procedures (5 main types across all sites) may limit the model’s ability to generalize to pain expressions from procedures not represented in training. The neonatal procedures are particularly homogeneous, focusing on common NICU interventions. Future work should expand to include broader pain etiology diversity including post-operative pain, chronic disease-related pain, and different types of acute injuries.

Additionally, our annotation protocol required pain ratings every 10 seconds during neonatal recordings, which may have influenced infant behavior. The frequent verbal interaction with caregivers during rating sessions could potentially have altered crying patterns or facial expressions compared to completely unobtrusive monitoring. This represents an important ecological validity consideration for clinical deployment, where truly passive monitoring would occur without any intervention.

Third, cultural factors and individual differences in pain expression present challenges for generalizable automated assessment. Research documents substantial cultural variation in pain expression, with some cultures encouraging emotional expression while others value stoicism [27]. Our dataset predominantly comprises Western participants from North American medical centers, potentially limiting generalizability to other cultural contexts. Individual personality differences also influence expression, with extroverted individuals typically showing more overt pain behaviors than introverted individuals experiencing identical noxious stimuli [16].

### Temporal modeling limitations

Our use of independent 3-second temporal windows represents a significant architectural limitation that may miss important pain dynamics. Pain experience often evolves across longer timescales, with anticipatory anxiety building before painful procedures, acute pain surges during tissue manipulation, and gradual recovery phases that unfold over minutes to hours [18,38]. By treating each 3-second segment independently, AVPENet cannot capture these longer-term temporal patterns, potentially missing clinically important information about pain trajectories.

This limitation manifests in several ways. First, the model cannot distinguish between different temporal pain patterns with identical momentary expressions—for instance, a brief pain spike versus sustained high pain cannot be differentiated if expressions in individual 3-second windows are similar. Second, important contextual information from preceding moments is lost; a grimace occurring after several minutes of calm may have different clinical significance than one following sustained distress, but our model treats them identically. Third, anticipatory behaviors preceding painful events (such as increased anxiety before procedure start) are not integrated into predictions, despite their clinical relevance for pain assessment [16].

We selected 3-second windows based on several practical considerations: computational tractability (longer windows dramatically increase memory requirements), annotation feasibility (human raters found rating shorter segments more reliable), and alignment with existing pain assessment tools that emphasize momentary behavioral states [13,11]. However, we acknowledge this represents a compromise that prioritizes practical implementation over optimal temporal modeling.

Future architectures should incorporate hierarchical temporal modeling that processes information at multiple timescales simultaneously. For example, a two-stream approach could analyze 3-second windows for fine-grained behavioral detection while a parallel recurrent network models longer-term context over 30–60 second periods. Alternatively, temporal transformer architectures could learn relevant temporal dependencies directly from data without pre-specifying window sizes [24,49]. Table 11 presents preliminary ablation results exploring different window durations.

**Table 11:** Temporal Window Duration Ablation Study.

Window Duration	MAE	PCC	Params (M)	Observations
1 second	1.12	0.82	32.7	Insufficient context; misses sustained patterns
2 seconds	0.97	0.86	32.7	Improved but still limited
**3 seconds**	**0.89**	**0.89**	**32.7**	**Optimal balance (selected)**
5 seconds	0.91	0.88	32.7	Diminishing returns; annotation difficulty
10 seconds	0.94	0.87	32.7	Performance degrades; conflicting behaviors within window

Note: All models use identical architecture with only window duration varied. Performance measured on same test set with ground truth aggregated over corresponding window lengths. The 3-second window provides optimal tradeoff between capturing sufficient behavioral context and maintaining temporal resolution for clinically relevant changes.

The ablation results confirm that 3 seconds represents a local optimum for our architecture and dataset, but they also highlight the fundamental limitation: performance plateaus regardless of window length because the architecture processes windows independently. True temporal modeling would require architectural changes (e.g., RNN/LSTM layers, temporal attention across windows) rather than simply extending window duration [48].

Fourth, the audio-visual modalities we employed cannot fully capture pain experience. Physiological signals including heart rate variability, blood pressure changes, cortisol elevation, and electrodermal activity provide complementary pain information less subject to voluntary control than behavioral displays [21]. Body movement, posture changes, and protective behaviors also signal pain but were not captured in our face-focused recordings.

Fifth, distinguishing pain from other negative emotions remains challenging with behavioral signals alone. Fear, anxiety, anger, and frustration can produce facial expressions and vocalizations similar to pain expressions [25]. While our ground truth annotation attempted to isolate pain from other distress sources using contextual information, some confounding likely remains. This limitation suggests that behavioral assessment should be complemented by contextual information about pain sources, patient history, and current clinical situation.

## Future research directions

The present work opens numerous avenues for future investigation. Multimodal expansion represents an immediate priority, integrating physiological signals such as heart rate, respiratory rate, blood pressure, electrodermal activity, and stress hormones with behavioral assessment [21,44]. Physiological signals provide complementary information less subject to voluntary modulation, potentially improving accuracy and robustness particularly for patients who suppress behavioral expressions.

Contextual information integration could substantially improve assessment by disambiguating pain from other negative emotions and calibrating predictions based on patient-specific factors. Electronic health record data including diagnosis, current medications, recent procedures, baseline pain levels, and individual pain sensitivity could inform personalized pain models [11,18]. Procedure timing information could distinguish anticipatory anxiety from procedural pain from post-procedure recovery.

Temporal modeling improvements could capture pain dynamics across multiple timescales. Hierarchical architectures could process short-timescale behavioral events (individual cries, facial expressions) while simultaneously modeling longer-timescale pain trajectories spanning minutes to hours [24]. Change detection algorithms could flag sudden pain increases indicating treatment failure or complications.

Personalization and adaptation represent critical directions for clinical deployment. Current practice employs a single population-average model for all patients, ignoring individual differences in expression. Patient-specific calibration using brief labelled data from each individual could adapt the model to personal expression patterns [31,47]. Meta-learning approaches could enable rapid adaptation to new patients with minimal labelled data.

Interpretability and explainability research could enhance clinical trust and adoption. Beyond attention visualization, concept-based explanations could identify interpretable mid-level features (specific facial action units, cry characteristics) driving predictions [30,40]. Counterfactual explanations could show how changing specific inputs would alter predictions, helping clinicians understand model reasoning.

Expanded population validation could assess generalisability beyond the neonatal and adult populations studied here. Paediatric populations spanning infancy through adolescence express pain differently across developmental stages [12]. Geriatric populations with age-related changes in facial mobility may require special consideration [27]. Cross-cultural validation across diverse geographic regions and ethnic groups is essential for global applicability [19].

### Broader impact and ethical considerations

Automated pain assessment technologies carry significant potential benefits but also raise important ethical considerations. Recent reviews emphasize the ethical imperative of developing AI-based pain assessment while ensuring fairness, transparency, and appropriate clinical integration [60,65,66]. On the positive side, objective consistent pain measurement could reduce disparities in pain treatment that currently disadvantage women, racial minorities, and vulnerable populations [19,20]. Research documents systematic undertreatment of pain in these groups, partly attributable to biased interpretation of behavioral pain signals. Automated assessment based solely on objective behavioral patterns could potentially reduce such bias, though careful validation across demographic groups is essential.

However, algorithmic bias remains a serious concern. If training data underrepresents certain demographic groups or systematically differs in ground truth labeling across groups, the resulting model may exhibit differential performance. Our preliminary cross-racial analysis showed no significant bias, but larger-scale validation is essential [19,35]. Even unbiased average performance may mask individual-level errors, where specific patients receive systematically inaccurate assessments due to atypical expression patterns.

Privacy and data security considerations are paramount given the sensitive nature of pain-related audio-visual recordings. Participants must understand how their data will be used, stored, and shared, with particular attention to protecting identifiable recordings [43]. De-identification of stored data, encryption during transmission, access controls limiting who can view recordings, and automatic deletion policies minimize privacy risks. However, perfect de-identification of video and audio is challenging, as faces and voices provide strong identifying information.

The potential for misuse or inappropriate reliance on automated assessment requires consideration. Ethical stewardship requires continuous monitoring for algorithmic bias and unintended consequences [61]. Automated scores should support rather than replace clinical judgment, providing additional objective information that clinicians integrate with broader assessment [11,18]. However, busy clinical environments may incentivize over-reliance on automated scores without appropriate critical evaluation. Appropriate use policies, education about system limitations, and maintaining ultimate clinical authority over treatment decisions are essential safeguards.

#### Automation bias and clinical deskilling.

The deployment of automated pain assessment systems raises important concerns about automation bias—the tendency of human decision-makers to over-rely on automated recommendations even when they may be incorrect [61]. If clinicians begin to trust automated pain scores without maintaining critical evaluation skills, they may miss atypical pain presentations or fail to notice when the system produces erroneous assessments. This risk is compounded by the potential for *deskilling*, where reduced practice in manual pain assessment could erode clinicians’ ability to recognize subtle pain cues independently of automated systems.

Moreover, *model drift*—gradual degradation of model performance over time as clinical practices, patient populations, or recording equipment evolve—represents a serious concern. Our model was trained on data from 2022-2024; as clinical protocols change, new medical devices are introduced, or patient demographics shift, the model’s accuracy may silently decline without obvious warning signs. Continuous monitoring of model performance in deployment, periodic retraining with contemporary data, and maintaining human expertise as the ultimate authority for pain assessment are essential safeguards against these risks.

To mitigate these concerns, we recommend: (1) implementing automated pain scores as *decision support* rather than automated decision-making, always requiring clinician review; (2) establishing ongoing performance monitoring with regular audits comparing automated scores to expert human assessment; (3) maintaining regular training for clinical staff in manual pain assessment to preserve critical evaluation skills; and (4) developing alert systems that flag when model confidence is low or when predictions deviate significantly from expected patterns.

## Conclusion

This work presents a comprehensive multimodal deep learning framework for continuous pain intensity estimation via audio-visual fusion of non-speech vocalizations and facial expressions. Through rigorous evaluation on 3,247 carefully annotated segments from 428 subjects spanning neonatal and adult populations, we demonstrate that bidirectional cross-modal attention fusion achieves mean absolute error of 0.89 on a 0–10 pain scale, representing substantial improvements of 39% over audio-only and 28% over visual-only approaches. The demonstrated generalization across age groups (MAE 0.94 for neonates, 0.84 for adults), robustness to acoustic noise and facial occlusions, and real-time processing capability (34ms per segment) support practical feasibility of clinical deployment.

The architectural innovations presented here, particularly bidirectional cross-modal attention mechanisms learning complementary audio-visual representations, contribute methodological advances applicable beyond pain assessment to multimodal behavioral analysis more broadly. Recent comprehensive reviews of multimodal machine learning in healthcare demonstrate the growing maturity of these approaches [23,63–64]. The demonstrated value of appropriate pretraining (32.6% performance improvement), composite loss functions incorporating domain knowledge (23% improvement from ordinal consistency), and systematic ablation analysis provide a template for developing robust multimodal systems from modest-sized clinical datasets. The comprehensive evaluation framework including cross-population validation, robustness testing under realistic noise and occlusion conditions, and failure mode analysis establishes standards for rigorous assessment of clinical AI systems.

While important limitations remain—including ecological validity concerns about controlled data collection, challenges distinguishing pain from other negative emotions using behavioral signals alone, and questions about generalization to underrepresented cultural groups—this work represents significant progress toward objective automated pain assessment for vulnerable populations unable to self-report. The potential clinical applications including continuous monitoring in neonatal ICUs, post-surgical pain tracking, emergency triage support, and standardized assessment in chronic pain clinics could substantially improve pain management and patient outcomes if deployed thoughtfully with appropriate safeguards. Digital health tools have demonstrated sustained impact on pain management over 25 years, providing a foundation for integrating automated assessment technologies [62]. Our longitudinal tracking demonstration showed close alignment with patient self-reports (mean difference 0.32) while providing continuous measurements between sparse manual assessments, directly addressing a critical gap in current clinical practice.

The comparison with human performance benchmarks reveals that AVPENet approaches or exceeds the inter-rater reliability typical of trained clinical observers (ICC 0.87 versus 0.82 for human-human agreement), while providing perfect consistency where identical inputs always yield identical outputs. This level of performance, combined with interpretable attention visualizations revealing that the model learns clinically meaningful patterns (emphasizing brow/eye regions during high pain, coordinating audio peak intensity with facial grimacing), suggests genuine readiness for clinical translation studies. The demonstrated robustness to moderate acoustic noise (13.5% degradation at 20 dB SNR typical of clinical environments) and common facial occlusions (18% degradation with surgical masks) further supports real-world feasibility.

Future work should focus on several key priorities to advance clinical translation. First, prospective clinical trials are essential to validate that improved pain measurement translates to better clinical outcomes including enhanced pain control, reduced adverse events from undertreated pain, and improved patient satisfaction. These trials should examine implementation in specific clinical contexts (neonatal ICU, post-surgical recovery, emergency triage) where automated continuous monitoring addresses clear unmet needs. Second, expansion to include physiological signals (heart rate variability, electrodermal activity) and contextual information (procedure timing, medication history, patient demographics) could substantially enhance accuracy and specificity, particularly for distinguishing pain from other negative affective states. Third, larger-scale validation across diverse populations including multiple cultural groups, age ranges spanning early childhood through geriatric populations, and various pain types (acute procedural, chronic disease-related, neuropathic) is necessary to ensure fairness, generalizability, and equitable performance across all patient groups [35].

Fourth, interpretability research could enhance clinical trust through concept-based explanations identifying specific facial action units and acoustic features driving predictions, counterfactual explanations showing how input changes would affect outputs, and uncertainty quantification providing confidence estimates that alert users when predictions are unreliable. Fifth, thoughtful consideration of ethical implications must accompany technical development. Recent comprehensive reviews emphasize the ethical imperative of ensuring fairness, transparency, and appropriate clinical integration of AI-based pain assessment systems [60,65,66]. This includes rigorous assessment and mitigation of potential algorithmic bias across demographic groups [61], privacy-preserving architectures that minimize retention of identifiable audio-visual data, appropriate use policies that maintain clinical judgment authority while leveraging automated insights, and ongoing post-deployment surveillance monitoring for performance degradation or unintended consequences.

In conclusion, multimodal audio-visual fusion via bidirectional cross-modal attention offers a promising approach to objective pain assessment in non-verbal patients. The demonstrated performance improvements (approaching human inter-rater reliability while eliminating observer variability), architectural innovations (learned multimodal consistency checking through attention mechanisms), comprehensive evaluation (age-stratified validation, robustness testing, interpretability analysis), and practical deployment considerations (real-time processing, graceful degradation under noise/occlusion, computational efficiency) provide a foundation for continued development toward clinical translation. With appropriate attention to rigorous clinical validation, fairness assessment across diverse populations, and ethical deployment policies that prioritize patient welfare and maintain human oversight, automated pain assessment could become a valuable tool supporting clinicians in delivering optimal pain management to all patients—particularly those most vulnerable and least able to advocate for themselves.

This work demonstrates that machine learning, properly designed and rigorously evaluated, can augment human clinical expertise in addressing one of medicine’s most persistent challenges: accurately recognizing and appropriately treating pain in those who cannot speak for themselves. The path forward requires continued collaboration between computer scientists, clinicians, ethicists, and patients to ensure that technological advances serve the fundamental goal of reducing suffering and improving care for all.
